# Tripartite split-GFP assay to identify selective intracellular nanobody that suppresses GTPase RHOA subfamily downstream signaling

**DOI:** 10.3389/fimmu.2022.980539

**Published:** 2022-08-18

**Authors:** Laura Keller, Claudine Tardy, Laetitia Ligat, Soazig Le Pennec, Nicolas Bery, Faten Koraïchi, Patrick Chinestra, Mélissa David, Rémi Gence, Gilles Favre, Stéphanie Cabantous, Aurélien Olichon

**Affiliations:** ^1^ Centre de Recherche en Cancérologie de Toulouse (CRCT), Université de Toulouse, Institut National de la Santé et de la Recherche Médicale (INSERM), Centre National de la Recherche Scientifique (CNRS), Université Toulouse III-Paul Sabatier, Centre de Recherches en Cancérologie de Toulouse (CRCT), Toulouse, France; ^2^ Laboratoire de Biologie Médicale Oncologique, IUCT-Oncopôle, Toulouse, France; ^3^ Le Pôle Technologique du Centre de Recherches en Cancérologie de Toulouse, Plateau de Protéomique, Toulouse, France; ^4^ Institut National de la Santé et de la Recherche Médicale (INSERM), Unité Mixte de Recherche (UMR) 1188 Diabète athérothrombose Réunion Océan Indien (DéTROI), Université de La Réunion, Saint Denis de La Réunion, France

**Keywords:** RHOA GTPase, tripartite split-GFP, nanobodies, RHO-ROCK signaling, single domain antibody (sdAb)

## Abstract

Strategies based on intracellular expression of artificial binding domains present several advantages over manipulating nucleic acid expression or the use of small molecule inhibitors. Intracellularly-functional nanobodies can be considered as promising macrodrugs to study key signaling pathways by interfering with protein-protein interactions. With the aim of studying the RAS-related small GTPase RHOA family, we previously isolated, from a synthetic phage display library, nanobodies selective towards the GTP-bound conformation of RHOA subfamily proteins that lack selectivity between the highly conserved RHOA-like and RAC subfamilies of GTPases. To identify RHOA/ROCK pathway inhibitory intracellular nanobodies, we implemented a stringent, subtractive phage display selection towards RHOA-GTP followed by a phenotypic screen based on F-actin fiber loss. Intracellular interaction and intracellular selectivity between RHOA and RAC1 proteins was demonstrated by adapting the sensitive intracellular protein-protein interaction reporter based on the tripartite split-GFP method. This strategy led us to identify a functional intracellular nanobody, hereafter named RH28, that does not cross-react with the close RAC subfamily and blocks/disrupts the RHOA/ROCK signaling pathway in several cell lines without further engineering or functionalization. We confirmed these results by showing, using SPR assays, the high specificity of the RH28 nanobody towards the GTP-bound conformation of RHOA subfamily GTPases. In the metastatic melanoma cell line WM266-4, RH28 expression triggered an elongated cellular phenotype associated with a loss of cellular contraction properties, demonstrating the efficient intracellular blocking of RHOA/B/C proteins downstream interactions without the need of manipulating endogenous gene expression. This work paves the way for future therapeutic strategies based on protein-protein interaction disruption with intracellular antibodies.

## Introduction

Recombinant antibody technology has so far provided genetically encoded high affinity reagents for common immunological assays used in fundamental and applied research (1–3). Peculiar applications such as tracing or modulating intracellular proteins in living cells are ascribed to special antibody fragments or alternative scaffolds that rely on single chain binding domains (4). Among antibody fragments that can be used inside the cell, nanobodies have emerged as promising molecular tools (5, 6). For instance, nanobodies from immunized animals or from synthetic scaffolds were efficiently engineered to specifically recognise not only an ectopic protein such as the Green Fluorescent Protein (GFP) (7, 8), or several linear epitope tags (9, 10), but also endogenous proteins (11, 12). In this regard, intracellular antibodies (also called intrabodies) present the advantage to disrupt endogenous protein functions either by functionalization through targeted protein degradation (13), ER-rerouting (14), or by competing with endogenous protein partners, thus offering an alternative strategy to small molecule inhibitors.

Proteins of the RHOA-subfamily (RHOA, RHOB and RHOC isoforms) are small GTPases belonging to the RAS superfamily. Likewise, these proteins behave like molecular switches. Upon stimuli, Guanine nucleotide Exchange Factors (GEFs) activate a fraction of RHOs (more than 95% of the cellular RHO proteins are present in the inactive GDP-bound state (15, 16)), by promoting their GTP loading. This nucleotide exchange results in a conformational change of the small GTPase, that is recognized by so-called effector proteins and triggers downstream cellular response. RHOA-subfamily small GTPases regulate key signalling pathways implicated in cell division, cell motility or other cellular processes (17, 18) and RHOA notably controls the ROCK/acto-myosin pathway that drives F-actin fiber formation and subcellular region contractility (19–21). The dysregulations of their expression and/or activity have been associated with several diseases including multiple cancers (22, 23), vascular or neurological disorders (24, 25).

The global inhibition of RHO proteins is currently achieved by RNA interference or by the use of the ADP-ribosylating bacterial exoenzyme C3 (26, 27). RNA interference can induce compensation mechanisms with other RHO proteins, making the resulting cellular phenotypes difficult to interpret with this strategy (13, 28). The ADP-ribosylating bacterial exoenzyme C3 irreversibly modifies RHO proteins leading to their subsequent degradation. Moreover, some small molecule inhibitors have been developed to prevent RHO activation, however by targeting a limited set of GEF (29). Therefore, there is an unmet need for a strategy that could specifically inhibit the active GTP-bound forms of RHO proteins, without perturbation of RHO expression or RAC activities. We hypothesized that intrabodies preferentially recognizing the conformational active state of these proteins thereby competing with endogenous effector binding, could be an efficient strategy. In this line, we previously generated from a synthetic phage display library (NaLi-H1) based on a unique nanobody scaffold, several conformational intrabodies that preferentially bind the conformational state loaded with GTP of the RAS-related RHO GTPases RHOA or RHOB (13, 30, 31). However, the clones so far selected which showed potential blocking activities did not present enough selectivity towards the RHOA subfamily over the close RAC1 subfamily, and one clone that preferentially recognised RHOB was non-blocking without being functionalized through a domain that recruits a multicomponent E3-ligase catalytic activity.

Here we present a more selective GTP-bound RHO nanobody, isolated from the synthetic nanobody library NaLi-H1, with RHOA subfamily blocking properties (31). After a competitive phage display strategy designed to enrich the library towards RHOA-GTP, we performed a phenotypic screening based on actin fiber loss associated with immunoprecipitation assays to identify a new nanobody. We further determined its intracellular selectivity as well as its effector blocking mechanism by using the tripartite split-GFP sensitive protein-protein interaction assay (32, 33). We then demonstrated that this nanobody interferes with the RHOA/ROCK actomyosin pathway, leading to a phenotypic switch from a rounded to elongated phenotype with impaired contractility. Overall, such nanobody appeared as an original and efficient tool to inhibit intracellularly GTPase activities in normal cells or in diverse pathological models.

## Material and methods

### Plasmids and lentiviral vectors

RHO GTPases and nanobodies were expressed as recombinant proteins from bacterial expression vectors or from mammalian expression vectors. 2SHA-RHO mutants tagged with the twin Streptag II (IBA) were expressed from a pET vector as previously described (31). Nanobodies from the NaLi-H1 library, referred to as hs2dAb for humanized synthetic single domain antibody, are expressed in a pHEN- hs2dAb-6his-myc-PIII phagemid. Hs2dAb were subcloned NcoI/NotI into the intrabody expression plasmid pIB-GFP or pIB-mCherry or pIB-IRES-MTSmCherry (13, 30, 31). Other intracellular nanobody expressing plasmids, pTRIP-TRE-Ib-myc-IRES-BFP, pIb-myc and pIb-myc-IRES-BFP, were also previously described (13). For periplasmic expression, hs2dAb-6his-myc insert was digested from pIB-GFP and inserted in a modified pHEN6-VHH-6his, thus creating a periplasmic expression vector pHEN6-hs2dAb-6his-myc-6his.

Specific plasmids were constructed for the tripartite split-GFP assays. For carboxy-terminal GFP11 tag fusions, pIb-hs2dAb-6hismyc-GFP was digested AgeI and Acc65I to remove the GFP and replaced with a PCR product encoding a Glycine Serine flexible linker followed by a carboxy-terminal GFP11 tag. For RHO-GTPases, the pGFP10-RHOA expressing CA or DN RHOA, RAC1 or CDC42 mutants were generated by site-directed mutagenesis and cloned into BspeI/XbaI sites of pcDNA_GFP10-Nter fusion vector previously described (34). For the pGFP11-RBD, the Rho Binding Domain of Rhotekin was amplified by PCR from pGST-RBD pGEX (Addgene#15247) and inserted into NotI/ClaI cloning sites of pcDNA_GFP11-Cter fusion vector previously described in 19. A pGFP11-RBD-TRE-GFP10-RHOA lentiviral vector co-expressing 10-RHOA and RBD-11 was generated by subcloning the pTRE tight RBD-11 cassette into the MluI site of an HIV-1-based lentiviral pTrip- vector carrying a tetracycline response element (TRE) (BIVICplatform, IFR 150, CHU Rangueil, Toulouse).

### Cell lines, transfection method and reagents

HeLa (cervical adenocarcinoma), MRC5-SV (human immortalized fibroblasts), WM266.4 (metastatic melanoma) cell lines (ATCC) were grown in DMEM (Lonza^®^) supplemented with 10% FCS at 37°C in a humidified incubator with 5% CO_2_. Transient transfection of DNA plasmids was performed using the Jet Prime method, as indicated by the supplier (PolyPlus Transfection^®^).

Western Blots were probed with the following antibodies: mouse monoclonal 26C4 anti-RHOA (1/500, O/N, 4°C, Santa Cruz Biotechnology^®^), goat polyclonal anti-myc tag HRP conjugated (1/3000, 1 hour, Room Temperature RT, Novus Biologicals^®^), mouse monoclonal anti-RAC1 (1/1000, O/N, 4°C, Millipore^®^), Ser19 P-Myosin light chain (1/500, O/N, 4°C Cell Signaling Technology^®^), GAPDH (1/2000, O/N, 4°C, Cell Signaling Technology^®^), mouse monoclonal Chitin Binding Domain (1/1000, O/N, 4°C NEB Biosciences^®^). Anti-myc tag (clone 9E10) used in immunofluorescence and flow cytometry experiments was a gift from S. Moutel. Anti-GFP10 rabbit polyclonal antibodies were obtained after rabbit immunization with synthetic peptides corresponding to GFP10 (DLPDDHYLSTQTILSKDLN) (Millegen, France^®^). Detection was performed using peroxidase-conjugated secondary antibodies and chemiluminescence detection kit (Biorad^®^) except for Ser19 P-Myosin light chain which was revealed with Amersham ECL Prime Western Blotting Detection Reagent^®^.

The production of stable cell lines with tetracycline inducible (Tet-on) hs2dAb expression was performed with lentiviral technology. The p-Ib-myc-IRES-BFP lentivirus was produced according to the tri-transfection procedure using the plasmids pTRIP-TRE-Ib-myc-IRES-BFP, pLvPack and pLvVSVg (Sigma^®^) in 293T cells for viral production. WM266.4 were previously transduced with the rtTA doxycycline-inducible transactivator, and then cells were further transduced with the IB-IRES-BFP lentivirus containing supernatant.

In order to establish the most homogeneous cell lines, for transduction efficiency, 24h after doxycycline induction, cells were then sorted on a BD Influx™ cell sorter for their cytoplasmic BFP fluorescence intensity. Flow cytometry data were analyzed with Kaluza software (Beckman Coulter^®^).

### Subtractive phage display panning for isolating RHO-GTP specific hs2dAb

The NaLi-H1 library of humanized synthetic single domain antibody (31) was used for this study. A subtractive panning protocol was designed to isolate hs2dAb selective for the RHOA-GTP-Chitin binding domain from chitinase A1 (CBD) or twin StrepTag (2S) fusion of RHOA GTPase active mutant (RHOA L63). Constructions were expressed transiently during 24 hours in HEK293 cells and captured freshly after cell lysis on magnetic beads before incubation with the library phages. Chitin magnetic beads (NEB^®^) or StrepTactin coated magStrep HC (IBA^®^) beads were used. A phage display panning alternating rounds on chitin beads with rounds on StrepTactin beads was performed during 4 rounds. From the second round of panning, a depletion step on GDP-loaded wild type RHOA or N19 inactive mutant and on RHOB L63, RHOC L63, RAC1 L61 active mutants was included. The adequate amount of antigen coated beads was incubated for 2 hours with the phage library (10^13^ phages diluted in 1 mL of PBS + 0.1% Tween 20 + 2% non-fat milk). Phages were previously adsorbed on empty streptavidin-coated magnetic beads (to remove nonspecific binders). Phages bound to streptavidin-coated beads or chitin beads were recovered on a magnet. Beads were washed 10 times (round 1), 15 times (round 2) and 25 times including long washes of one hour (rounds 3 and 4) with PBS-Tween 0.1%. Bound phages were eluted using triethylamine (Sigma Aldrich^®^) and E. coli (TG1 strain) were infected with the eluted phages. For rounds 2, 3 and 4, only 10^12^ phages were used as input.

### Pull down assays

Co-precipitations of intracellular nanobodies (myc-tagged hs2dAb) with CA RHOA mutants were performed after transient co-transfection of pCBD-RHOA L63 with pIb-myc in HeLa cells. After 24 hours, cleared cell lysates containing CBD-RHO mutants in buffer (50 mM Tris pH 7.4. 500 mM NaCl, 10 mM MgCl2, 1% TritonX100, protease and phosphatase inhibitors supplemented) were incubated with chitin beads (NEB Biolabs^®^) for 1 hour at 4°C. Co-precipitation was revealed by RHOA antibody and myc antibody.

Co-precipitations of intrabodies (myc-tagged hs2dAb) with endogenous mammalian RHO proteins were performed after doxycycline induction of pIb-myc-IRES-BFP in WM266.4 cells. Cleared cell lysates were then incubated with His tag purification beads (Roche^®^) during 45 minutes at 4°C for Ni-NTA IMAC pull down. Beads were then washed thrice in washing buffer (Tris pH 7.4 50 mM, NaCl 150 mM, MgCl2 10 mM, 0.1% Tween20), and immunoprecipitates were then analyzed by Western blotting and revealed by RHOA antibody and myc antibody.

### Immunofluorescence staining

Transfected cells grown on coverslips were fixed in 3.7% paraformaldehyde and permeabilized with PBS-Triton 0.1%, blocked with PBS-BSA 8%, incubated with primary antibody mouse monoclonal anti-myc tag (9E10 clone 1/800, O/N, 4°C) then Pacific blue mouse secondary antibodies (1/400, 1 hour, RT, BD Bioscience^®^). Alexa 568-Phalloidin (1/40, 1 hour, RT, Invitrogen^®^) was used to reveal actin stress fibers. All coverslips were mounted in Mowiol. Data acquisition was carried out on a Zeiss Axiovert inverted microscope and figure montage using Image J.

### In cell interaction using the tripartite split-GFP assay

MRC5 cells expressing the GFP1-9 fragment (GFP1-9_MRC5) were cotransfected with plasmids expressing either GFP10 tagged constitutively active RHOA L63, RAC1 L61 or CDC42 L61 mutants or inactive RHOA N19 mutant, and with GFP11 tagged hs2dAb, RBD or PAK-BD. 20 hours after transfection, cells were fixed with 3.7% PFA (Sigma-Aldrich^®^) and permeabilized with 0.1% triton X100 (Sigma-Aldrich^®^). Cells were subsequently co-stained with 9E10 anti-myc monoclonal (1/3000, 4°C, 1h30min) antibody and with anti-GFP10 fragment antibody (1/1000, 4°C, 1h30min). Secondary antibodies were respectively mouse APC and rabbit Pacific blue (1/100, 1H, 4°C, BD Biosciences^®^) (1/200, 1H, 4°C BD Biosciences^®^). GFP fluorescence was measured using FACS MACS Quant 10 cytometer^®^. At least 20,000 gated events were counted for each sample and analysed using Kaluza analysis software (Beckman Coulter^®^). GFP fluorescence was measured using FACS MACS Quant 10 cytometer^®^. The geometric mean from the GFP channel was determined from the gating region corresponding to double GFP10 and GFP11 positive labelling that correlate to GFP10 RHO mutants and hs2dAb expression levels respectively.

The GFP fluorescence was also imaged on a Zeiss Axiovert inverted microscope. For immunofluorescence experiment, transfected cells were fixed in 3.7% paraformaldehyde and permeabilized with PBS-Triton 0.1%, blocked with PBS-BSA 8%, incubated with primary antibody mouse monoclonal anti-myc tag (9E10 clone 1/800, O/N, 4°C) and anti-GFP10 fragment antibody (1/1000, O/N, 4°C) then respectively with Pacific blue mouse secondary antibodies (1/400, 1 hour, RT, BD Bioscience^®^) and Alexa 568-anti mouse secondary antibody (1/400, 1 hour, RT, BD Bioscience^®^).

For intracellular competition experiments, hs2dAb N-terminally fused to mCherry were co-transfected with a tet-on inducible bidirectional promoter vector expressing GFP10-RHOAWT and GFP11-RBD in MRC5_GFP1-9 cells, a cell line referred to as triSFP-RHOA. 20 hours after transfection, GFP10-RHOAWT and GFP11-RBD expression were induced with doxycycline. 16 hours later, GFP fluorescence and hs2dAb expression were measured using FACS MACS Quant VYB cytometer. At least 20,000 gated events were counted for each sample and analysed using FlowJo analysis software.

### Recombinant protein expression and purification

RHO GTPase production. 2SHA-RHO were expressed in BL21 *E.coli* cells from a pET vector. Transformed bacteria cells were used to grow 3mL LB-carbenicillin (100 µg/ml) cultures overnight at 37°C prior to inoculation in baffled flasks containing 1 L of the same media. Cells were allowed to grow at 37°C until OD600 reached 0.5-0.7. Cells were then induced with IPTG at a final concentration of 100 µM and grown for an additional 20 hours at 25°C. Cells were harvested by centrifugation at 4000g for 20 min at 4°C. The pellets were re-suspended in lysis buffer (50 mM Tris HCl, pH 8, 150 mM NaCl, 5 mM MgCl2, 0.1% triton, 1 mM DTT, lysozyme and DNase I 1X, protease inhibitors) and lysed by sonication on ice prior to centrifugation (30 min, 15000 g, 4°C). StrepTactin SuperFlow Plus (IBA^®^) matrix was equilibrated in buffer A (50 mM Tris HCl, pH 8, 150 mM NaCl, 5 mM MgCl2) and was incubated with supernatant for 2 hours at 4°C. Then supernatant and matrix were loaded on a simple column in order to maximise capture of 2SHA-RHO proteins. Matrix was washed by 15 mL of washing buffer (300 mM Nacl, 50 mM tris pH8, 5 mM MgCl2, 0.1% tween20). RHO proteins were then eluted in buffer A containing 10 mM Biotin (Sigma^®^). Dialysis was performed overnight against buffer A containing 15% glycerol.

Nanobody purification. Hs2dAb were produced in XL1blue E.coli grown in TB-ampicillin (100 µg/mL) medium supplemented with 1% glucose in the start culture and 0.1% glucose during induction with 1 mM IPTG. After overexpression for 16h at 28°C, the cells were harvested, suspended in 15 mL ice-cold TES (Tris 100 mM pH 8, EDTA 1 mM, Sucrose 500 mM) and stored at -80°C. 30 mL of a ¼ dilution of TES buffer was added to the re-suspended pellets prior to vortex briefly and to keep for 30 min at 4°C. After centrifugation (30min, 13000g, 4°C), the periplasmic extract containing hs2dAb was purified by affinity chromatography. The protein extract was incubated 2 hours in the presence of His-Tag purification beads (Roche^®^) previously equilibrated with equilibration buffer (12 mM Tris pH8, 0.125 mM EDTA, 65 mM Sucrose, 300 mM NaCl, 10 mM Imidazole pH7). Beads were washed with 30 ml of washing buffer (10 mM Tris pH8, 150 mM NaCl, 10 mM Imidazole pH7). Hs2dAb were then eluted with elution buffer (500 mM Imidazole pH7, 25 mM Tris pH6.8, 300 mM NaCl) and dialysis was performed for 16 hours at 4°C in PBS 10% Glycerol. OD at 280 nm was measured in order to determine hs2dAb concentration.

### Affinity measurement

Hs2dAb binding studies based on SPR technology were performed on BIAcore T200 optical biosensor instrument (GE Healthcare^®^). Capture of recombinant 6xHis tagged hs2dAb, expressed in XL1blue and purified as previously reported (35) was performed on a nitrilotriacetic acid (NTA) sensor chip in HBS-P+ buffer (10 mM Hepes pH 7.4, 150 mM NaCl, and 0.05% surfactant P20) (GE Healthcare). The four flow cells (Fc) of the sensor chip were used: one (Fc 1) to monitor nonspecific binding and to provide background corrections for analyses and the other three flow cells (Fc 2, 3, and 4) containing immobilized 6xHis tagged hs2dAb for measurement.

For immobilization strategies, flow cells were loaded with nickel solution (10 μL/min for 60 s) in order to saturate the NTA surface with Ni^2+^ and an extra wash was done using running buffer containing 3mM EDTA after the nickel injection. His-tagged hs2dAb in running buffer was injected in flow cells at a flow-rate of 10 μL/min. Total amount of immobilized hs2dAb was 250-300 resonance units. (RUs; 1 RU corresponds approximately to 1 pg/mm2 of protein on the sensor chip). A Single-Cycle Kinetics (SCK) analysis to determine the dissociation equilibrium constant (K_D_) was carried out. SCK method prevents potential inaccuracy due to sensor chip regeneration between cycles which are necessary in the conventional Multiple Cycle Kinetics (MCK) (36). SCK binding parameters are evaluated for each injection according to the tools and fit models of the BIAevaluation software, giving similar values than MCK. As hs2dAb were smaller proteins than their respective antigens, hs2dAb were captured on the sensor chip then the recombinant GTPases were used as analytes and were injected sequentially with increased concentrations ranging between 3.125 nM to 50 nM in a single cycle without regeneration of the sensor chip between injections. Binding parameters were obtained by fitting the overlaid sensorgrams with the 1:1. Langmuir binding model of the BIAevaluation software version 1.0.

### ELISA and G-LISA assays

For ELISA detection of RHO GTPases, wells of StrepTactin-coated plates (IBA^®^) were coated with 100 nM of recombinant CA or DN RHOA, CA RAC1 or CA CDC42 mutants as 2S-HA fused proteins (200 µl in TBS by well) during 2 hours at RT and then blocked with 5% milk in TBS-Tween 0.05% (blocking buffer) for 1 hour at RT. Several dilutions of hs2dAb in blocking buffer were applied to the ELISA plates in duplicates for 1 hour at RT. Next, we added 1 µg/ml anti-myc HRP antibody (QED Biosciences, 18824P^®^) in blocking buffer for 1 hour at RT and the reaction was visualized by the addition of 100 μl chromogenic substrate (Thermoscientific^®^, 1-step ultraTMB, 34028) for 1 min. The reaction was stopped with 50 μl H_2_SO_4_ 1N and absorbance at 450 nm was measured using FLUOstar OPTIMA microplate reader. Plates were washed three times with washing buffer (TBS containing 0.05% (v/v) Tween 20) after each step. All steps are performed under agitation (400 rpm).

To perform competition experiments using the G-LISA^®^ procedure, we used G-LISA^®^ RHOA (BK124) and RAC1 (BK128) assays (Cytoskeleton) according to the manufacturer’s instructions to assess if purified hs2dAb were able to compete with RBD or PAK domain. We pre-incubated 10-fold serial dilutions of NR53, RH12 or RH28 for 1 hour with 2S-HA RHOA or RAC1 before performing the G-LISA assays. Hs2dAb-6His-myc-6his inputs were controlled in an ELISA after capture on nickel coated plates as described previously (15).

### Quantitative RT-PCR

RH28 and NR27 WM266.4 cells were harvested after 18 and 24 hours of induction with 1µg/ml Doxycycline, and RNA was extracted following RNeasy Plus minikit (Qiagen) procedure. RNA concentration was measured with Nanodrop. Reverse transcription was carried out on 1 μg of RNA using RT iScript kit (Biorad). Priming for reverse transcription was done with combined oligo(dT) and random hexamers.

Quantitative PCRs were performed on cDNA using iQ SyBr Green kit (Biorad) on a ViiA-7 RT-PCR system (Applied Biosystems). RHOA transcript was quantified according to the standard 2^−ΔΔCt^ method after normalization to *B2M* (beta 2-microglobulin).

Primers used for determination of RHOA transcript are the following: RHOA_sens: 5’-TGGAAGATGGCATAACCTGTC and RHOA_anti-sens 5’- AACTGGTGGCTCCTCTGG; B2M_sens: 5’-ACCCCCACTGAAAAAGATGA and B2M _anti-sens 5’-ATCTTCAAACCTCCATGATG

### Cell culture in a 3D matrix

Cells were embedded in a 3D matrix constituted of collagen type I (1.5 mg/ml, Corning^®^) in EMEM (Eagle’s Minimal Essential Medium; 2 ×, Lonza^®^) at a concentration of 1,5 × 10^5^ cells/ml. Drops (30 μl) were placed for 1 hour upside down at 37 °C to allow solidification of the matrix. The complete medium was then added and hs2dAb expression was induced by doxycycline. 6 hours later, cell morphology was observed under a Nikon inverted microscope and drops were harvested, collagenase I (100 U/mL final concentration, ThermoFisher^®^) added and cells centrifuged. Pelleted cells were then lysed in RIPA buffer containing phosphatase and protease inhibitors.

### Gel contraction assay

A total of 0.5M cells were embedded in a 3D matrix constituted of collagen type I (3.1 mg/ml, Corning^®^) in EMEM (Eagle’s Minimal Essential Medium; 2 ×, Lonza) and plated in a 24-wells plate. After one hour of polymerisation at 37°C, the gel was gently dissociated from the edge of the well with a 200µl-pipet tip and the expression of hs2dAb induced by adding doxycycline to the cell culture medium. The plates were scanned after 72 hours and the area of the gel and the plate were measured and quantified with ImageJ software. For each well, the percentage of gel contraction was calculated using the formula 100 − [(area of the gel/area of an empty well) × 100].

### Statistical analysis

Reported values represent mean ± standard deviation (SD) of at least three independent experiments. Unless otherwise stated, student paired t-tests were performed for comparison with GraphPad Prism 9. *, p<0.05; **, p<0.01; ***, p<0.001; ****, p< 0.0001.

## Results

### Phenotypic screening selection of RHOA-GTP blocking intrabodies

The goal of the present study was the selection of a blocking intrabody more selective towards RHOA subfamily. After four rounds of phage display against RHOA in its GTP-bound conformation (see methods), 30 ELISA-positive families of clones were isolated (**Supplementary Figure 1**), including new sequences as well as a few copies of the previously identified RH12 (RHO binder H12). To screen for intracellular binders of RHOA-GTP family, this novel set of GTP-bound RHO nanobodies was subcloned into a mammalian expression vector with carboxy terminal dual 6xHis and myc tags as reporters of expression. Their ability to bind active RHOA conformation while expressed as intracellular nanobodies was first evaluated by a chitin bead co-immunoprecipitation assay after co-transfection with a CA RHOA (constitutively active RHOA L63) mutant bearing a C-terminal CBD (Chitin Binding Domain) tag (**Supplementary Figure 1**). We focused on 8 clones that were efficiently immunoprecipitated with the CA RHOA. As negative controls, we chose, among a set of nanobodies originating from previous phage display selections towards non-related protein targets, two clones referred to as NR27 and NR53 (31).

In order to identify a RHOA subfamily activity-blocking intrabody, we performed a phenotypic screen based on actin F-fiber staining after transfection of the nanobody candidates in HeLa cells. Indeed, in adherent cells, RHO inhibition mediated by the ADP-ribosylating bacterial exoenzyme C3 (26, 27) or after expression knockdown (37, 38) induces actin fiber loss and a dramatic morphological change. As a control of effective RHO inhibition, we treated HeLa cells with the tat-C3 exoenzyme and observed in more than 50% of the cells a stretched cellular shape with a retracted cytoplasm and elongated protrusions (**Supplementary Figure 2**). Among the 8 positive CA RHOA binders, 3 clones, referred to as RH28, RH29 and RH35, induced a stretched and shrunken cellular phenotype as shown in cells stained with a homogenous pattern of nanobodies expression detected in HeLa cells (**Supplementary Figure 2**). This cellular shape appeared similar to the one induced by recombinant tat-C3 treatment. In contrast, in NR-expressing control cells or cells with no detectable expression of RH28, RH29 or RH35 nanobodies, actin F staining revealed organisation in fibers. Therefore, we hypothesized that these 3 nanobodies might potentially be positive hits for blocking the active RHO signalling pathway.

### The tripartite split-GFP assay demonstrates the intracellular biochemical selectivity of the selected intrabodies

**Figure 1:**
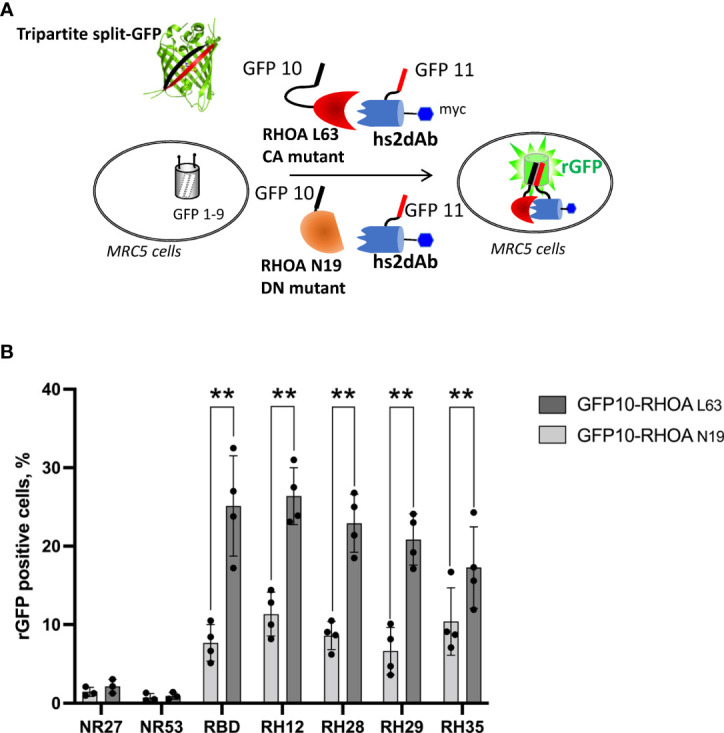
The tripartite split GFP assay demonstrates the intracellular interaction and the selective recognition of the active RHO conformation by the selected intrabodies. **(A)** Principle of the tripartite split-GFP complementation assay adapted to assess hs2dAb/RHO interaction. β-strand 10 (GFP10) and β-strand 11 (GFP11) are fused to RHOA mutants (either DN RHOA N19 or CA RHOA L63) and hs2dAb, respectively. These constructions are transfected in a MRC5 cell line that constitutively expresses the detector fragment GFP1–9 (β-strands 1–9). When protein interaction occurs, GFP10 and GFP11 strands are tethered and then spontaneously associate with GFP1–9 fragment to form a full-length GFP. If the two proteins do not interact, GFP10 and GFP11 are not tethered and entropy is too high to allow complementation with GFP1–9. **(B)** Percentage of reconstituted GFP (rGFP) fluorescent cells analyzed by flow cytometry for the indicated transfection conditions. P-values were calculated using a Student’s t test. **, p<0.01.

We first confirmed the intracellular interaction between the 3 selected clones and the active RHOA conformation using the tripartite split-GFP protein-protein interaction reporter system (32). The tripartite split-GFP reporter assay was selected for its high sensitivity due to its irreversibility and lack of background fluorescence. In this assay, a GFP variant gene is separated in three parts: the β-strand 10 (GFP10) is fused to one partner of the interaction, the β-strand 11 (GFP11) fused to the second partner, and the remaining amino-terminal β-strand 1 to 9 (GFP1-9) acts as a detector moiety of the interaction, leading to the formation of a reconstituted GFP (rGFP) that becomes fluorescent after chromophore maturation (**Figure 1A**). The split-GFP system was previously successfully implemented to monitor active RHO or RAS GTPase interactions with their respective effector domains in cells (34). Here, the nanobodies were fused to the GFP-11 tag in amino-terminal while the CA RHOA mutant was carboxy-terminally fused to the GFP10 fragment. These constructions were expressed in a human fibroblast MRC5 cell line already validated for its stable and homogenous expression of the GFP1-9 (34). After 24 hours of co-transfection within the MRC5_GFP1-9 cell line, the expression of each moiety and rGFP fluorescence was quantified. As a positive control of intracellular RHO interaction, we used the RHOTEKIN effector RHO Binding Domain (RBD), fused to a C-terminal GFP11 tag (34). Its co-transfection with CA GFP10-RHOA led to 25% of rGFP positive cells (**Figure 1B** and **Supplementary Figure 3**). Similar percentages of rGFP positive cells were also detected in cells co-expressing the CA GFP10-RHOA for the 3 potential RHO-GTP intrabodies (23%, 21% and 17% for RH28, RH29 and RH35 respectively). Control analysis of co-expression indicated similar levels of RHO mutants and the various nanobodies in all experiments (**Supplementary Figure 3**). In comparison, 1% of rGFP cells could be detected when co-expressing the CA GFP10-RHOA and the negative controls NR27 or NR53, demonstrating thereby the intracellular interaction between each of the 3 intrabodies and the active form of RHOA.

Flow cytometry quantification of the reconstituted GFP (rGFP) fluorescent signal demonstrated that the high affinity, already characterized, RH12 nanobody presented a similar signal intensity and selectivity towards the active RHOA conformation. (**Figure 1B** and **Supplementary Figure 3**). We also assessed the capability of the 3 hits to selectively recognize the active RHOA conformation in the intracellular environment by testing the interaction with a DN (Dominant Negative N19) mutant of RHOA (**Figures 1A, B** and **Supplementary Figure 3**). All of them appeared selective of the active RHOA conformation since significantly lower percentages of rGFP cells were observed with the DN of RHOA (23% vs. 8.6%; 21% vs 6.6%; 17% vs. 10% respectively).

To further confirm the intracellular selectivity, we expressed in *E.coli* and purified the three nanobodies and compared their binding affinities for RHOA mutants by SPR (**Supplementary Figure 4**). All of them had a K_D_ in the sub-nanomolar range for CA RHOA but no measurable binding of DN RHOA was observed, which is consistent with the results obtained *in-cellulo*. To assess whether these hits also bind the two other isoforms of the RHOA subfamily, RHOB and RHOC, we measured their K_D_ values for the CA mutants (**Supplementary Figure 4**). RH28, RH29 and RH35 displayed nanomolar range affinities for all members of RHOA subfamily which share more than 95% amino acid identities when excluding the carboxy-terminal hypervariable domain (39).

In this assay, we also evaluate the affinities towards the CA mutants of RAC1 GTPase and found that RH29 and RH35 nanobodies, but not RH28, could also bind RAC1 with equilibrium dissociation constant in the range of 10 to 20 nM (**Supplementary Figure 4**). This result suggested that RH28 is the only clone highly selective towards RHOA subfamily active proteins. RH28 selectivity towards the active RHOA conformation was additionally confirmed by measuring the capacity to detect RHO GTPases in ELISA. RH28 recognized the CA mutant of RHOA while no signal was observed with CA mutants of the phylogenetically closest RHO GTPase members of RHOA subfamily, RAC1 and CDC42 (**Supplementary Figure 5**).

**Figure 2:**
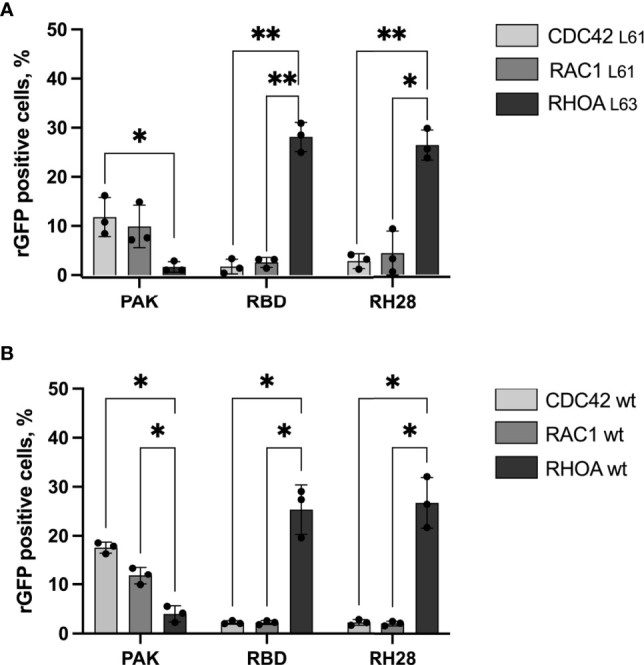
The RH28 is selective for active RHOA conformation in living cells. Percentage of reconstituted GFP (rGFP) fluorescent cells analyzed by flow cytometry for the indicated transfection conditions. **(A)** constitutively active mutants **(B)** wild-type proteins. P-values were calculated using a Student’s t test.*p<0.05; **p<0.01.

Considering potential discrepancies between *in vitro* measurements and biochemical interactions in the complexity of the intracellular environment, we assayed the selectivity of the RH28 among the 3 close subfamilies of RHO GTPases in cells by quantifying the rGFP signal obtained with RAC1 and CDC42, in their active state. As expected, the RBD tested with CA RAC1 or CA CDC42 led to a significantly lower amount of rGFP cells in comparison to the CA RHOA (28% vs. 2.6% and 28% vs. 1.7%), confirming that intracellularly, the RBD did not recognize the active forms of RAC1 nor CDC42. We validated the PAK domain as a positive control for RAC1 and CDC42 selective interaction since we observed a higher number of rGFP cells in comparison to RHOA (9.9% vs. 1.6% and 11.8% vs. 1.6%). Similarly to the natural RBD, the RH28 showed a clear selectivity towards CA RHOA and no cross reactivity with neither CA RAC1 nor CA CDC42 (26.4% vs. 4.4% and 26.4% vs. 2.8%) (**Figure 2A** and **Supplementary Figure 6**). We confirmed this result on the wild-type form of RHO GTPases (**Figure 2B** and **Supplementary Figure 6**). These results demonstrated that, in cells, the RH28 clone does not bind the closest GTPases related to RHOA even when they are expressed at a higher level than endogenous proteins. This result was also supported by the capacity of the myc tagged RH28 nanobody to immunoprecipitate the endogenous RHOA protein (data not shown). Collectively, these results led us to conclude that RH28 is an artificial biomolecular domain highly selective of the GTP-bound conformation of RHOA-like subfamily. We confirmed that the RH28 domain (produced in *E.coli* and purified) performed also in conventional RHOA immunoprecipitation assays after cytochalasin D or serum stimulation (data not shown).

### RH28 competes with RHOA subfamily effector binding

RHOA subfamily global inhibition was extensively studied in various cellular models using the tat-C3 exoenzyme or more selectively by RNA interference. In adherent cultured epithelial cells, the main phenotype is linked to the RHOA/ROCK pathway inhibition that lead to actomyosin contractility defect, actin fiber disorganization, and focal adhesion disassembly (40, 41). To assess if the phenotype associated with RH28 expression in the initial screen effectively reflects RHOA inhibition, we analyzed actin cytoskeleton in human fibroblast MRC5 cell line that displays high density of actin stress fibers in 2D cell culture. Phalloidin staining of cells expressing NR27 nanobody highlights the cellular shape surrounded by strong cortical actin fibers and a high density of stress fibers crossing throughout the cell. In contrast, RH28 expression abolished totally actin fibers and induced stretched elongated cells with only subtle actin staining decorating multiple protrusions at the periphery (**Supplementary Figure 7**), an effect that phenocopied RHOA/ROCK pathway inhibition upon either C3 or ROCK inhibitor treatments (42).

**Figure 3:**
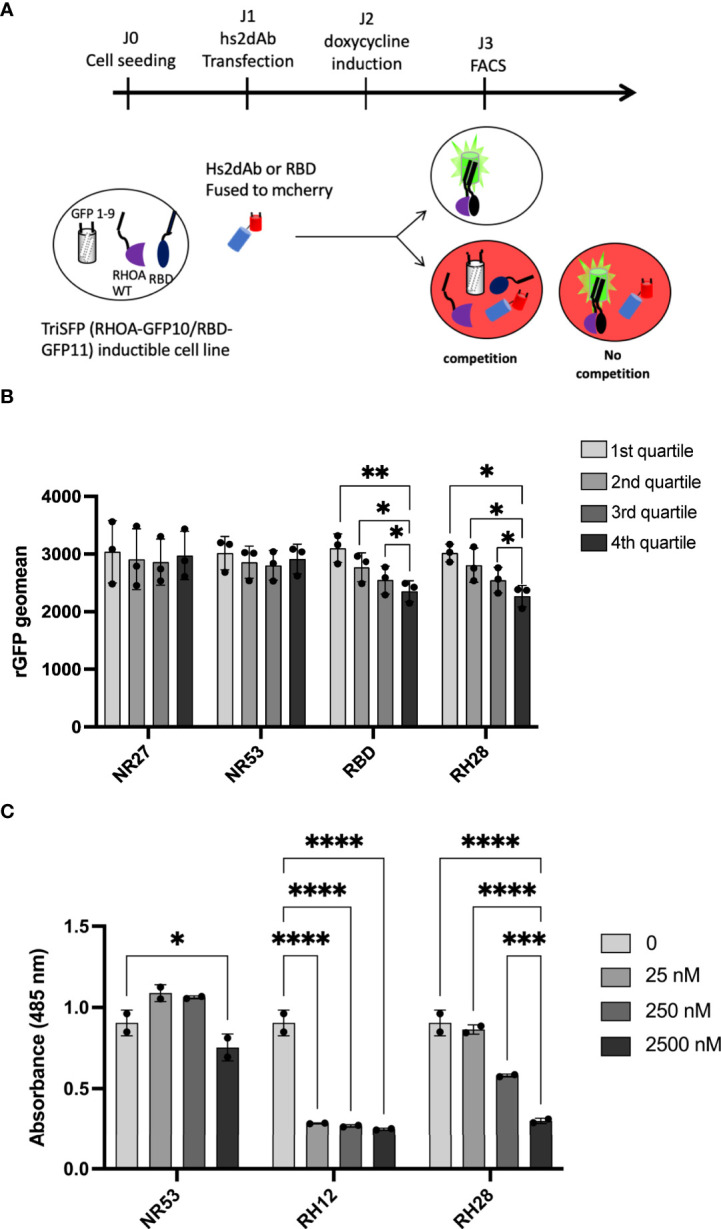
RH28 competes with RBD for active RHOA recognition. **(A)** Principle of the tripartite split-GFP complementation assay adapted to assess hs2dAb/RBD competition. Hs2dAb were transfected in the triSFP RHOA cell line, 24 hours before doxycycline induction. Among cells that express hs2dAb, a competition with RBD would lead to a decrease in the intensity of the rGFP fluorescence in mCherry positive cells. **(B)** For each hs2dAb, rGFP fluorescence intensity was quantified among the 4 different populations of mCherry positive cells (i.e., among the 4 cell populations ranked according to increasing levels of hs2dAb (or RBD) expression). **(C)** RHOA G-LISA competition assay with 10-fold dilutions of hs2dAb. Results were analyzed with two-way ANOVA model. Absorbance at 485 nm reflects RHOA-GTP captured by the coated RBD. P-values were calculated using a Student’s t test. *p < 0.05; **p < 0.01; ***p < 0.001; ****p < 0.0001.

To evaluate whether the RH28-mediated actomyosin perturbation was effectively linked with RHOA inhibition, we tested the RH28 capacity to compete with CA RHOA and the RHOTEKIN RBD interaction in the triSFP RHOA activation reporter cells, a cell line previously generated to sense RHOA activity with the tripartite split-GFP assay (34). In this cellular model (MRC5_GFP1-9 expressing the CA GFP10-RHOA and GFP11-RBD under the control of doxycycline), we transiently transfected mCherry fusions of different nanobodies and the RBD (**Figure 3A**). Flow cytometry quantification of the rGFP fluorescence level, corresponding to CA RHOA and RBD interaction amount within each cell, was performed 48 hours after intrabody transfection in mCherry positive cells. We compared the rGFP fluorescence intensity among the different quartiles of mCherry expression levels in transfected cells (**Figure 3B** and **Supplementary Figure 8A**). As expected, we observed a decrease in rGFP fluorescence reaching 20% between the lowest and the highest quartile (rGFP geomean in mCherry last quartile 2356 vs. 3103 in first quartile) when RBD-mCherry was transiently transfected (**Figure 3B**). Indeed, in this assay, RBD-mCherry was supposed to behave as a direct competitor of CA-GFP10-RHOA and GFP11-RBD interaction. In the RH28-mCherry condition, we also observed significant rGFP fluorescence decrease in a similar range (rGFP geomean in mCherry last quartile 2272 vs. 3020 in first quartile) despite the fact that RH28-mCherry expression appeared lower than the RBD-mCherry (**Supplementary Figure 8A**). By contrast, the non-RHO nanobodies NR27 and NR53 did not affect rGFP fluorescence even for the highest expression levels in the 4^th^ quartile, which confirms that they do not compete with RHOA activity and that the dose-dependent decay induced by RH28 is associated with its binding properties. This result suggests that the RH28 may efficiently impede the GFP11-RBD binding to CA-GFP10-RHOA, similarly to the RBD itself.

To confirm that the binding site of the RH28 interferes with the effector binding domain, we set up an *in vitro* competition assay based on the G-LISA RHO activity assay (**Figure 3C** and **Supplementary Figure 8B**). The G-LISA assay is based on the capture of GTP-bound RHO by RBD-like proteins covalently linked to the surface of the well. Like RH12, already reported as depleting the GST-RBD pull down assay (30), RH28 preincubation with 25 nM of recombinant CA 2SHA-RHOA induced a significant decrease of the signal in a concentration dependent manner (**Figure 3C**). We confirmed that no competition was observed on the RAC1 G-LISA assay (**Supplementary Figure 8C**). Altogether, these results suggest that the RH28 sterically impairs the RBD binding interface of RHOA proteins and might be a competitor of RHO effectors.

### RH28 intrabody efficiently blocks RHOA/ROCK pathway in melanoma cancer cells

**Figure 4:**
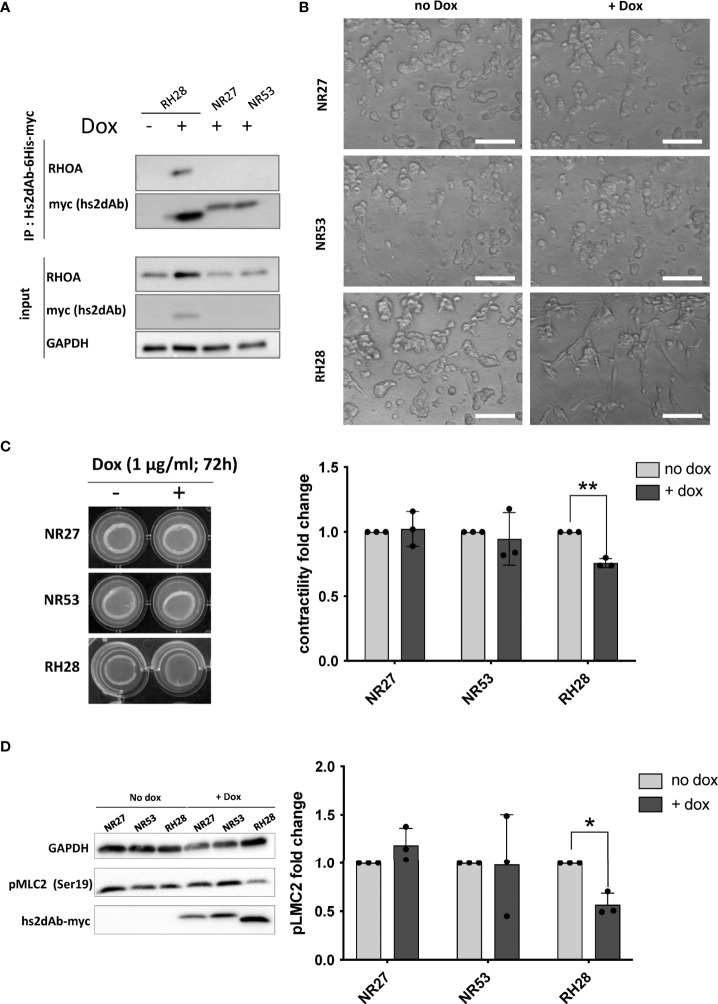
RH28 intrabody efficiently inhibits actomyosin contractility and blocks RHOA/ROCK pathway in melanoma cancer cells. **(A)** RH28 selectivity validation in WM266.4 lentiviral cell line. Expression of RH28 and NR-hs2dAb was induced or not with doxycycline at 1µg/ml. After 20 hours of induction, cells were harvested and cleared cell lysates were incubated with Ni-NTA beads for 45 min. Endogenous RHOA proteins was revealed with corresponding antibodies and hs2dAb were revealed with myc-tag antibody. **(B)** Representative images of cell line morphology in 3D collagen drops after 6 hours of hs2dAb expression. WM266.4 cell lines were seeded in collagen and phenotypes were analyzed 24 hours post doxycycline induction. At 40X magnification, elongated or rounded cell shape could be observed. Scale bar: 50 µm. **(C)** Representative images and quantification of gel contraction after 72 hours of treatment by doxycycline. **(D)** Representative immunoblot and quantification analysis of myosin light chain phosphorylation (pMLC2) status in the 3 different cell lines seeded in collagen drops. P-values were calculated using a Student’s t test.*p<0.05; **p<0.01.

We finally assessed if the blocking properties of the RH28 intrabody could disturb cellular phenotype or functions. In melanoma, high level of actomyosin contractility due to RHOA/ROCK pathway has been associated with amoeboid migration of melanoma cell lines (43) as well as resistance to shear forces during extravasation (44). In this model, in order to have a better control over the expression level of intrabodies, we produced lentiviral cell lines expressing, under the control of doxycycline inducible promoter, a bicistronic gene encoding both the hs2dAb-6his-myc and a BFP fluorescent reporter through an IRES. After setting up the dose response to doxycycline to express similar level of RH28 and of the NR27 control we confirmed the functionality of the RH28 expressed in this model through RHOA immunoprecipitation experiments (**Figure 4A**). An increase in RHOA protein level could be observed upon RH28 expression that cannot be solely explained at the mRNA level (**Supplementary Figure 9A**), suggesting a potential stabilisation of RHOA induced by the RH28 nanobody. We further confirmed that the RH28 did not recognize RAC1 in this model (**Supplementary Figures 9B, C**). Moreover, upon RH28 expression, we observed in 2D cell culture an elongated phenotype that reminds fibroblast stretching previously observed (**Supplementary Figure 7**). In order to study the cellular behaviour in 3D environment in which RHO activity plays a more important role than in 2D (45, 46), cells were seeded in collagen drops. In contrast to their normal spread morphology in 2D, WM266.4 exhibited as expected a rounded phenotype in this 3D matrix (44, 47). In comparison to NR controls and to non-induced doxycycline condition, the RH28 expression uniformly induced a striking switch from rounded cells to highly elongated cells (**Figure 4B**). This phenotype was also associated with a defect in actomyosin contractility (24% decrease upon RH28 expression) as demonstrated by an impaired collagen retraction (**Figure 4C**). To confirm RHOA/ROCK pathway inhibition, we analyzed the expression of phosphorylated cofilin and phosphorylated Myosin Light Chain (pMLC) which is one canonical target defining ROCK signalling activity (48). Upon RH28 expression induction in cells grown in 3D collagen drops, elongated cell phenotype was associated with a significant decrease of phospho-MLC2 amount (**Figure 4D**). All together, these results demonstrate that RH28 functions in an intracellular context by blocking RHOA downstream signalling through a direct interference with RHOA-GTP effectors, leading to a specific cellular phenotype.

## Discussion

Small GTPases of RHOA subfamily are master regulators of cellular processes involving actomyosin dynamics, such as cell division, cell migration or invasion. In this study we identified and characterized the nanobody RH28 as an intrabody selective towards the GTP-bound conformation of RHOA with no apparent cross reactivity towards RAC1 or CDC42. The RH28 behaves as an artificial RHO Binding Domain combining the high intracellular stability of nanobodies with inhibitory properties. This tool opens opportunities to investigate the fine-tuning of RHOA subfamily activation homeostasis in various biological contexts.

We previously generated from a synthetic phage display library based on a unique nanobody scaffold, several intracellular molecular binders of the GTP-bound RHOA or RHOB conformers (13, 30, 31). The lack of selectivity of such molecular tools targeting RHO GTPase activities or pathways is a key point in the interpretation of the cellular response. Actually, the highly conserved G domains that switch conformation between GDP or GTP loading led to the identification, in our previous work, of several conformational nanobodies. While expressed as intracellular antibodies, some hs2dAb were not able to block GTPase signalling while others were efficient blocking-nanobodies but cross-reacted with the RAC subfamily of GTPases, the close homologues of the RHOA subfamily. One pan RHO/RAC-GTP nanobody appeared inert at a moderate expression level and was engineered as an active RHO BRET biosensor (30). Another nanobody functionalised with a Fbox, referred to as F-B6 (13), was efficiently targeting RHOB-GTP for protein degradation, albeit through the dependency of the fused domain that requires a multicomponent E3-ligase catalytic activity which is not controlled by the tool itself. Of note, the nanobody referred to as RH12 with subnanomolar affinity towards RHO/RAC-GTP (31), displaces the endogenous effectors Rho Binding Domain in biochemical assays (30), induces a complex phenotype of cell border shrinkage and further toxicity that impaired cell viability (30, 31). While this nanobody mediated signalling blockade appeared encouraging to develop a macrodrug to alter RHO-GTP function, the lack of selectivity between RHOA-like and RAC1 subfamilies impeded its development as these two major RHO GTPases display opposite functions in many cellular contexts (22, 49–51).

It is challenging to translate the biochemical selectivity of molecular interaction of proteins or antibodies assessed *in vitro* to their behaviour in the intracellular complexity. Intracellular functionality of domain antibodies or alternative scaffolds have been assessed by numerous approaches, such as fluorescent two-hybrid (52) or BRET (30, 53) assays which allow, due to their reversibility, a dynamic quantification of the protein-protein interaction. However, these approaches show inherent background signal that may require tight expression control of the two components. Here, we used the tripartite split-GFP protein-protein interaction reporter assay because no signal background could emanate from the three components. Its main advantages are the absence of false positive interacting partners, and the ability to reveal low affinity interactions due to the irreversibility of the reconstituted GFP (32, 33). Moreover, the split-GFP system was previously successfully implemented to monitor active RHO or RAS GTPase interactions with their respective effector domains in cells (34). The lack of signal obtained for the RH28 with RAC1 or CDC42 active mutants thus asserts that this nanobody could not cross react with these GTPases. Nevertheless, among positive signals, this assay does not reflect conventional binding kinetic parameters of partners’ interactions. It is noteworthy that the irreversibility of the rGFP induces accumulation of a signal to a certain extent, thus revealing low affinity, transient interactions, or strong interaction with a similar level of quantification. Accordingly, we observed, in previous development of the assay (32, 34) or here with the RH12 and the RBD example, that a low signal discrepancy in this assay reflects a high selectivity quantified in a reversible biochemical interaction assay such as ELISA or SPR (15). Actually, although the selectivity of the RH12 towards the active conformation of RHOA GTPases was quantitatively much higher than the one of the RHOTEKIN RBD using *in-vitro* biochemical assays (15), this strong selectivity discrepancy was eclipsed in the tripartite split-GFP measurements. Therefore, we considered here that the binding of RH28, RH29 and RH35, reflected a strong conformational selectivity towards the active form of RHOA in cells. We also analysed cross-reactivity with RAC1 and CDC42 both *in vitro* and in cells. Although we did not formally assess the interaction with the 15 other members of RHO GTPase subfamilies, the lack of binding of the RH28 to the two RHOA-subfamily closest members may suggest that it only interacts with active RHOA/B/C proteins (39).

The phenotype induced by the RH28 expression in cells appeared more defined than the one resulting of the RH12 blockade (30, 31). Indeed, in HeLa, MRC5 or melanoma WM266-4 cell lines, cells expressing RH28 displayed a stretched elongated shape. We reasoned that this phenotype was probably correlated with the inhibition of the RHOA/ROCK pathway as the lack of contractility neither antagonizes the RAC1 mediated protrusion formation nor retracts the rear of migrating cells, thus elongating the cells by stretching them until adhesion collapsed. This phenotype was reminiscent of the ROCK inhibition by 10µM of Y27632 or other ROCK small molecule inhibitors (51), and we confirmed that this pathway was downregulated upon RH28 expression as phosphorylated Myosin Light Chain (pMLC) levels decreased. This marker of acto-myosin contraction is involved in mechanisms related to cancer cell plasticity between amoeboid or mesenchymal phenotypes, which implies different motile behaviours. Recently, high level of melanoma cell plasticity was demonstrated as a feature of MAPKi-therapy resistant melanoma (54). Several studies reported the association of RHOA or RHOB GTPases with invasive cancer resistance (54–56). ROCK inhibitors have been reported in preclinical studies to impair migration and invasion (57), to potentiate the immune system (58) or sensitize the immune checkpoint blockade response (59). Albeit the RHOA/ROCK pathway is actively targeted with numerous ROCK pharmacological inhibitors with a prospect to block invasion and metastasis in clinical trials (60), to date, none of them are approved for clinical use in cancer therapy. This may account to the lack of selectivity of ATP binding pocket kinase inhibitors that often induce side effects. The advantage of a nanobody that blocks RHOA-GTP downstream pathway may reside in the exquisite selectivity of antibody binding interface. However, the main challenge of biomolecular drugs that target intracellular activities remains their delivery as recombinant protein or mRNA inside tumor cells (61). 

## Data availability statement

The raw data supporting the conclusions of this article will be made available by the authors, without undue reservation.

## Author contributions

Conception and design: LK, GF and AO. Development of methodology: LK and AO. Acquisition of data (including facilities): LK, CT, LL and AO. Analysis and interpretation of data: LK, CT, SC, GF and AO. Administrative, technical, or material support: FK, NB, SLP, RG, PC, SC, LL and MD. Study supervision: GF, SC and AO. Financial support: GF. All authors revised and agreed with the submission of the manuscript.

## Funding

This work was financially supported by the grant Fondation pour la Recherche Médicale (FRM) (Equipe labellisée FRM [DEQ20170839117]). NB was supported by the Fondation pour la Recherche Médicale (FRM, FDT20130928310). This study used some fundings received under a collaboration contract with Cisbio Bioassays.

## Acknowledgments

We acknowledge the Pôle Technologique du CRCT – Plateau Imagerie, Cytométrie and Vectorologie for assistance in cell sorting and lentiviral production. We thank P. Pellehaut for comments on the manuscript. We thank the grant Fondation pour la Recherche Médicale (FRM) (Equipe labellisée FRM [DEQ20170839117]).

## Conflict of interest

Authors NB, LK, GF and AO are co-inventors on the patent PTC/EP2016/052136, concerning the discovery of RHO-GTP single-domain antibodies and their applications.

The authors declare that this study received funding from Cisbio Bioassays. The funder was not involved in the study design, collection, analysis, interpretation of data, the writing of this article or the decision to submit it for publication

## Publisher’s note

All claims expressed in this article are solely those of the authors and do not necessarily represent those of their affiliated organizations, or those of the publisher, the editors and the reviewers. Any product that may be evaluated in this article, or claim that may be made by its manufacturer, is not guaranteed or endorsed by the publisher.

## References

[1] Hoogenboom HR. Selecting and screening recombinant antibody libraries. Nat Biotechnol (2005) 23:1105–16. doi: 10.1038/nbt1126 16151404

[2] Helma J Cardoso MC Muyldermans S Leonhardt H. Nanobodies and recombinant binders in cell biology. J Cell Biol (2015) 209(5):633–44. doi: 10.1083/jcb.201409074 PMC4460151 26056137

[3] Pillay TS Muyldermans S. Application of single-domain antibodies (“Nanobodies”) to laboratory diagnosis. Ann Lab Med (2021) 41(6):549–58. doi: 10.3343/alm.2021.41.6.549 PMC8203438 34108282

[4] Kaiser PD Maier J Traenkle B Emele F Rothbauer U. Recent progress in generating intracellular functional antibody fragments to target and trace cellular components in living cells. Biochim Biophys Acta (2014) 1844(11):1933–42. doi: 10.1016/j.bbapap.2014.04.019 24792387

[5] Wagner TR Rothbauer U. Nanobodies right in the middle: Intrabodies as toolbox to visualize and modulate antigens in the living cell. Biomolecules (2020) 10(12):1701. doi: 10.3390/biom10121701 PMC7767433 33371447

[6] Gettemans J De Dobbelaer B. Transforming nanobodies into high-precision tools for protein function analysis. Am J Physiol-Cell Physiol (2021) 320(2):C195–215. doi: 10.1152/ajpcell.00435.2020 33264078

[7] Rothbauer U Zolghadr K Tillib S Nowak D Schermelleh L Gahl A. Targeting and tracing antigens in live cells with fluorescent nanobodies. Nat Methods (2006) 3(11):887–9. doi: 10.1038/nmeth953 17060912

[8] Tang JCY Szikra T Kozorovitskiy Y Teixiera M Sabatini BL Roska B. A nanobody-based system using fluorescent proteins as scaffolds for cell-specific gene manipulation. Cell (2013) 154(4):928–39. doi: 10.1016/j.cell.2013.07.021 PMC4096992 23953120

[9] Traenkle B Segan S Fagbadebo FO Kaiser PD Rothbauer U. A novel epitope tagging system to visualize and monitor antigens in live cells with chromobodies. Sci Rep (2020) 10(1):14267. doi: 10.1038/s41598-020-71091-x 32868807 PMC7459311

[10] Götzke H Kilisch M Martínez-Carranza M Sograte-Idrissi S Rajavel A Schlichthaerle T. The ALFA-tag is a highly versatile tool for nanobody-based bioscience applications. Nat Commun (2019) 10(1):4403. doi: 10.1038/s41467-019-12301-7 31562305 PMC6764986

[11] Lin Y Chen Z Hu C Chen ZS Zhang L. Recent progress in antitumor functions of the intracellular antibodies. Drug Discov Today (2020) 25(6):1109–20. doi: 10.1016/j.drudis.2020.02.009 32112969

[12] Messer A Butler DC. Optimizing intracellular antibodies (intrabodies/nanobodies) to treat neurodegenerative disorders. Neurobiol Dis (2020) 134:104619. doi: 10.1016/j.nbd.2019.104619 31669671

[13] Bery N Keller L Soulié M Gence R Iscache AL Cherier J. A targeted protein degradation cell-based screening for nanobodies selective toward the cellular RHOB GTP-bound conformation. Cell Chem Biol (2019) 26:1544–58. doi: 10.2139/ssrn.3188332 31522999

[14] Marschall ALJ Dübel S Böldicke T. Recent advances with ER targeted intrabodies. Adv Exp Med Biol (2016) 917:77–93. doi: 10.1007/978-3-319-32805-8_5 27236553

[15] Keller L Tardy C Ligat L Gilhodes J Filleron T Bery N. Nanobody-based quantification of GTP-bound RHO conformation reveals RHOA and RHOC activation independent from their total expression in breast cancer. Anal Chem (2021) 93(15):6104–11. doi: 10.1021/acs.analchem.0c05137 33825439

[16] Ren XD Schwartz MA. Determination of GTP loading on rho. Methods Enzymol (2000) 325:264–72. doi: 10.1016/S0076-6879(00)25448-7 11036609

[17] Etienne-Manneville S Hall A. Rho GTPases in cell biology. Nature (2002) 420(6916):629–35. doi: 10.1038/nature01148 12478284

[18] Ridley AJ. Rho GTPase signalling in cell migration. Curr Opin Cell Biol (2015) 36:103–12. doi: 10.1016/j.ceb.2015.08.005 PMC4728192 26363959

[19] Pertz O Hodgson L Klemke RL Hahn KM. Spatiotemporal dynamics of RhoA activity in migrating cells. Nature (2006) 440:1069–72. doi: 10.1038/nature04665 16547516

[20] Reffay M Parrini MC Cochet-Escartin O Ladoux B Buguin A Coscoy S. Interplay of RhoA and mechanical forces in collective cell migration driven by leader cells. Nat Cell Biol (2014) 16(3):217–23. doi: 10.1038/ncb2917 24561621

[21] Sahai E Ishizaki T Narumiya S Treisman R. Transformation mediated by RhoA requires activity of ROCK kinases. Curr Biol (1999) 9:136–45. doi: 10.1016/S0960-9822(99)80067-0 10021386

[22] Haga RB Ridley AJ. Rho GTPases: Regulation and roles in cancer cell biology. Small GTPases (2016) 7(4):207–21. doi: 10.1080/21541248.2016.1232583 PMC5129894 27628050

[23] Sahai E Marshall CJ. RHO-GTPases and cancer. Nat Rev Cancer (2002) 2:133–42. doi: 10.1038/nrc725 12635176

[24] Flentje A Kalsi R Monahan TS. Small GTPases and their role in vascular disease. Int J Mol Sci (2019) 20(4):E917. doi: 10.3390/ijms20040917 30791562 PMC6413073

[25] Kalpachidou T Spiecker L Kress M Quarta S. Rho GTPases in the physiology and pathophysiology of peripheral sensory neurons. Cells (2019) 8(6):E591. doi: 10.3390/cells8060591 31208035 PMC6627758

[26] Aktories K Mohr C Koch G. Clostridium botulinum C3 ADP-ribosyltransferase. Curr Top Microbiol Immunol (1992) 175:115–31. doi: 10.1007/978-3-642-76966-5_6 1628497

[27] Sahai E Olson MF. Purification of TAT-C3 exoenzyme. Methods Enzymol. (2006) 406:128–40. doi: 10.1016/S0076-6879(06)06011-3. 16472655

[28] Boulter E Garcia-Mata R Guilluy C Dubash A Rossi G Brennwald PJ. Regulation of rho GTPase crosstalk, degradation and activity by RhoGDI1. Nat Cell Biol (2010) 12:477–83. doi: 10.1038/ncb2049 PMC2866742 20400958

[29] Lin Y Zheng Y. Approaches of targeting rho GTPases in cancer drug discovery. Expert Opin Drug Discov (2015) 10(9):991–1010. doi: 10.1517/17460441.2015.1058775 26087073 PMC4824952

[30] Keller L Bery N Tardy C Ligat L Favre G Rabbitts TH. Selection and characterization of a nanobody biosensor of GTP-bound RHO activities. Antibodies (2019) 8:8. doi: 10.3390/antib8010008 PMC6640709 31544814

[31] Moutel S Bery N Bernard V Keller L Lemesre E de Marco A. NaLi-H1: A universal synthetic library of humanized nanobodies providing highly functional antibodies and intrabodies. eLife (2016) 5. doi: 10.7554/eLife.16228 PMC4985285 27434673

[32] Cabantous S Nguyen HB Pedelacq JD Koraïchi F Chaudhary A Ganguly K. A new protein-protein interaction sensor based on tripartite split-GFP association. Sci Rep (2013) 3:2854. doi: 10.1038/srep02854 24092409 PMC3790201

[33] Pedelacq JD Cabantous S. Development and applications of superfolder and split fluorescent protein detection systems in biology. Int J Mol Sci (2019) 20(14):E3479. doi: 10.3390/ijms20143479 31311175 PMC6678664

[34] Koraïchi F Gence R Bouchenot C Grosjean S Lajoie-Mazenc I Favre G. High-content tripartite split-GFP cell-based assays to screen for modulators of small GTPase activation. J Cell Sci (2018) 131(1):jcs210419. doi: 101242/jcs.210419 29192060 10.1242/jcs.210419 PMC5818064

[35] Olichon A Surrey T. Selection of genetically encoded fluorescent single domain antibodies engineered for efficient expression in escherichia coli. J Biol Chem (2007) 282(50):36314–20. doi: 10.1074/jbc.M704908200 17921141

[36] Trutnau HH. New multi-step kinetics using common affinity biosensors saves time and sample at full access to kinetics and concentration. J Biotechnol (2006) 124(1):191–5. doi: 10.1016/j.jbiotec.2006.01.006 16580083

[37] Bousquet E Mazieres J Privat M Rizzati V Casanova A Ledoux A. Loss of RhoB expression promotes migration and invasion of human bronchial cells *via* activation of AKT1. Cancer Res (2009) 69:6092–9. doi: 10.1158/0008-5472.CAN-08-4147 19602596

[38] Vega FM Fruhwirth G Ng T Ridley AJ. RhoA and RhoC have distinct roles in migration and invasion by acting through different targets. J Cell Biol (2011) 193(4):655–65. doi: 10.1083/jcb.201011038 PMC3166870 21576392

[39] Olson MF. Rho GTPases, their post-translational modifications, disease-associated mutations and pharmacological inhibitors. Small GTPases (2018) 9(3):203–15. doi: 10.1080/21541248.2016.1218407 PMC5927519 27548350

[40] Ridley AJ Hall A. The small GTP-binding protein rho regulates the assembly of focal adhesions and actin stress fibers in response to growth factors. Cell (1992) 70(3):389–99. doi: 10.1016/0092-8674(92)90163-7 1643657

[41] Uehata M Ishizaki T Satoh H Ono T Kawahara T Morishita T. Calcium sensitization of smooth muscle mediated by a rho-associated protein kinase in hypertension. Nature (1997) 389(6654):990–4. doi: 10.1038/40187 9353125

[42] Worthylake RA Burridge K. RhoA and ROCK promote migration by limiting membrane protrusions. J Biol Chem (2003) 278(15):13578–84. doi: 10.1074/jbc.M211584200 12574166

[43] Rodriguez-Hernandez I Cantelli G Bruce F Sanz-Moreno V. Rho, ROCK and actomyosin contractility in metastasis as drug targets. F1000Research (2016) 5:783. doi: 10.12688/f1000research.7909.1 PMC4856114 27158478

[44] Pinner S Sahai E. Imaging amoeboid cancer cell motility *in vivo* . J Microsc (2008) 231(3):441–5. doi: 10.1111/j.1365-2818.2008.02056.x 18754999

[45] Petrie RJ Gavara N Chadwick RS Yamada KM. Nonpolarized signaling reveals two distinct modes of 3D cell migration. J Cell Biol (2012) 197(3):439–55. doi: 10.1083/jcb.201201124 PMC3341168 22547408

[46] Riching KM Keely PJ. Rho family GTPases: Making it to the third dimension. Int J Biochem Cell Biol (2015) 59:111–5. doi: 10.1016/j.biocel.2014.11.007 PMC4344808 25478651

[47] Yin Z Sadok A Sailem H McCarthy A Xia X Li F. A screen for morphological complexity identifies regulators of switch-like transitions between discrete cell shapes. Nat Cell Biol (2013) 15(7):860–71. doi: 10.1038/ncb2764 PMC3712499 23748611

[48] Julian L Olson MF. Rho-associated coiled-coil containing kinases (ROCK): structure, regulation, and functions. Small GTPases (2014) 5:e29846. doi: 10.4161/sgtp.29846 25010901 PMC4114931

[49] Bustelo XR Sauzeau V Berenjeno IM. GTP-binding proteins of the Rho/Rac family: regulation, effectors and functions *in vivo* . BioEssays News Rev Mol Cell Dev Biol (2007) 29(4):356–70. doi: 10.1002/bies.20558 PMC1971132 17373658

[50] Norman JC Price LS Ridley AJ Koffer A. The small GTP-binding proteins, rac and rho, regulate cytoskeletal organization and exocytosis in mast cells by parallel pathways. Mol Biol Cell (1996) 7:1429–42. doi: 10.1091/mbc.7.9.1429 PMC275992 8885237

[51] Sahai E Marshall CJ. Differing modes of tumour cell invasion have distinct requirements for Rho/ROCK signalling and extracellular proteolysis. Nat Cell Biol (2003) 5:711–9. doi: 10.1038/ncb1019 12844144

[52] Zolghadr K Mortusewicz O Rothbauer U Kleinhans R Goehler H Wanker EE. A fluorescent two-hybrid assay for direct visualization of protein interactions in living cells. Mol Cell Proteomics MCP (2008) 7(11):2279–87. doi: 10.1074/mcp.M700548-MCP200 18622019

[53] Bery N Legg S Debreczeni J Breed J Embrey K Stubbs C. KRAS-specific inhibition using a DARPin binding to a site in the allosteric lobe. Nat Commun (2019) 10(1):2607. doi: 10.1038/s41467-019-10419-2 31197133 PMC6565726

[54] Orgaz JL Crosas-Molist E Sadok A Perdrix-Rosell A Maiques O Rodriguez-Hernandez I. Myosin II reactivation and cytoskeletal remodeling as a hallmark and a vulnerability in melanoma therapy resistance. Cancer Cell (2020) 37(1):85–103.e9. doi: 10.1016/j.ccell.2019.12.003 31935375 PMC6958528

[55] Calvayrac O Mazières J Figarol S Marty-Detraves C Raymond-Letron I Bousquet E. The RAS-related GTPase RHOB confers resistance to EGFR-tyrosine kinase inhibitors in non-small-cell lung cancer *via* an AKT-dependent mechanism. EMBO Mol Med (2017) 9(2):238–50. doi: 10.15252/emmm.201606646 PMC5286377 28003335

[56] Delmas A Cherier J Pohorecka M Medale-Giamarchi C Meyer N Casanova A. The c-Jun/RHOB/AKT pathway confers resistance of BRAF-mutant melanoma cells to MAPK inhibitors. Oncotarget (2015) 6(17):15250–64. doi: 10.18632/oncotarget.3888 PMC4558149 26098773

[57] Patel RA Liu Y Wang B Li R Sebti SM. Identification of novel ROCK inhibitors with anti-migratory and anti-invasive activities. Oncogene (2014) 33(5):550–5. doi: 10.1038/onc.2012.634 PMC3977753 23396364

[58] Teiti I Florie B Pich C Gence R Lajoie-Mazenc I Rochaix P. *In vivo* effects in melanoma of ROCK inhibition-induced FasL overexpression. Front Oncol (2015) 5:156. doi: 10.3389/fonc.2015.00156 26236689 PMC4500923

[59] Kim S Kim SA Nam GH Hong Y Kim GB Choi Y. *In situ* immunogenic clearance induced by a combination of photodynamic therapy and rho-kinase inhibition sensitizes immune checkpoint blockade response to elicit systemic antitumor immunity against intraocular melanoma and its metastasis. J Immunother Cancer (2021) 9(1):e001481. doi: 10.1136/jitc-2020-001481 33479026 PMC7825261

[60] McLeod R Kumar R Papadatos-Pastos D Mateo J Brown JS Garces AHI. First-in-Human study of AT13148, a dual ROCK-AKT inhibitor in patients with solid tumors. Clin Cancer Res (2020) 26(18):4777–84. doi: 10.1158/1078-0432.CCR-20-0700 PMC7611345 32616501

[61] Bannas P Hambach J Koch-Nolte F. Nanobodies and nanobody-based human heavy chain antibodies as antitumor therapeutics. Front Immunol (2017) 8:1603. doi: 10.3389/fimmu.2017.01603 29213270 PMC5702627

