# Supplementary material for: Tripartite split-GFP assay to identify selective intracellular nanobody that suppresses GTPase RHOA subfamily downstream signaling
Source: Front Immunol. 2022 Aug 18;13:980539. doi: 10.3389/fimmu.2022.980539 (PMC9433928; doi:10.3389/fimmu.2022.980539)

Supplementary Material

1 Supplementary Figures

Supplementary Figure 1: Flowchart of the selection for inhibitory RHOA intracellular nanobody candidates. (1) Scheme of the phage display principle illustrating the 4 rounds of enrichment, each cycle starting by displaying the cDNA library of nanobodies on phages, followed by affinity selection, and recovery of phages to produce a sub library. (**2**) Monoclonal characterization of bacterial cell lines expressing a single clone of nanobody were used to screen in ELISA RHOA-GTP selective hs2dAb. (**3-4**) Subcloning and expression in mammalian cells allowed co-precipitation experiments with constitutively active RHOA mutants, followed by **(5)** a phenotypic screening of actomyosin destabilization mediated by nanobody intracellular expression.

Supplementary Figure 2: Screening of RHOA signaling blocking intracellular nanobodies by an actin F immunofluorescence assay. HeLa cells expressing transiently either controls (NR) hs2dAb or RH (anti RHO-GTP) hs2dAb were fixed 20 hours post transfection and stained using anti myc tag antibody and Alexa 568 phalloidin to detect actin stress fibers. White arrows indicate the various phenotypes among transfected cells expressing controls or RHO-GTP nanobodies. Scale bar 20 µm.

Supplementary Figure 3: The tripartite split GFP assay demonstrates the intracellular interaction and the selective recognition of the active RHO conformation by the selected intrabodies.

(A): Representative images of GFP11-hs2dAb and GFP10-RHOA transfected cells and rGFP visualized by fluorescence microscopy. Nanobodies or RHOTEKIN RBD (11-Myc Nb) and RHOA mutant (10-RHOA L63 or N19) expression were assessed with a myc tag antibody (APC) and with an antibody against GFP10 strand (Pacific Blue), respectively. Scale bar 100 µm. **(B)**: Nanobody expression control in the MRC5_GFP1-9 cell line. Expression of selected hs2dAb was quantified by flow cytometry by fluorescence geometric mean of the anti-myc antibody. **(C)**: RHOA mutant expression control in the MRC5_GFP1-9 cell line. Expression of RHOA mutants L63 or N19 was quantified by flow cytometry by fluorescence geometric mean of the anti-GFP10 antibody.

Supplementary Figure 4: Affinity determination. Single Cycle Kinetics analysis were simultaneously performed on immobilized His fusion nanobodies (250-300 RU), with five injections of analytes (from top to bottom panels: RHOA L63, RHOA N19, RHOB L63, RHOC L63 or RAC1 L61) at 3.125nM, 6.25nM, 12.5nM, 25nM, and 50nM; Times of buffer exchange are indicated by arrows. Analytes injections lasted for 120s each and were separated by 10s dissociation phases. Off-rate constants were calculated from an extended dissociation period of 10 min following the last injection according to the single cycle kinetics method. Each sensorgram (expressed in RUs as a function of time in seconds) represents a differential response where the response on an empty reference channel (Fc1) was subtracted. The red curves correspond to the data and the black curves represent the fit done by the BIAevaluation software. On-rate (k_on, sec^{-1}), off-rate (k_off, $M.sec^{-1}$) and dissociation equilibrium constants (K_D in nanomolar nM) are indicated for each condition. The absence of interaction is indicated by NI.

Supplementary Figure 5: *In vitro* selectivity characterization of RH28 with an ELISA assay. CA or DN 2SHA-RHO, CA 2SHA-RAC1 and CA 2SHA-CDC42 mutants were coated on StrepTactin plates and 10-fold dilutions of bacterially-produced RH28 were incubated. RH28 was revealed with myc tag antibody.

Supplementary Figure 6: The split-GFP tripartite assay demonstrates the intracellular selective recognition of active RHOA conformation by the RH28 hs2dAb. Controls of main Figure 2.

(A) and (C): hs2dAb expression control in the MRC5_GFP1-9 cell line. Expression of selected hs2dAb was quantified by flow cytometry by fluorescence geometric mean of the anti myc tag antibody signal (APC). **(B) and (D)**: RHOA, RAC1 and CDC42 expression control in the MRC5_GFP1-9 cell line. Expression of RHOA or RAC1 wild type or mutants was quantified by flow cytometry by fluorescence geometric mean of the anti-GFP10 antibody (Pacific Blue). **(C) and (D)**: expression controls for WT conditions were only quantified once.

Supplementary Figure 7: RH28 blocks the RHOA subfamily signaling to actomyosin and induces loss of actin fibers in MRC5 cells. MRC5 fibroblasts expressing transiently either controls (NR) or RH28 hs2dAb-6xHis-Myc were fixed 20 hours post transfection and stained using anti myc tag antibody and Alexa 568 phalloidin to detect actin stress fibers. Arrows indicated cells devoid of actin fibers. Scale bar 20 µm.

Supplementary Figure 8: Competition between RH hs2dAb and the RBD in cells using the tripartite split-GFP and *in vitro* using G-LISA assay.

(A): mCherry positive cells were divided into four populations based on the quartile values of mCherry intensity geometric mean. mCherry geometric mean directly reflects the expression level of hs2dAb or RBD within cells. **(B) and (C):** Principle of RHOA and RAC1 G-LISA assays adapted to assess hs2dAb competition with RBD or PAK. **(D):** RAC1 G-LISA assay with 10-fold dilutions of hs2dAb. Results were analyzed with two-way ANOVA model. *, $p<0.05$; **, $p<0.01$; ***, $p<0.001$; ****, $p< 0.0001$

Supplementary Figure 9: RH28 intracellular nanobody selectivity towards RHOA in WM266-4 melanoma cells.

(A): Quantitative RT-PCR of RHOA transcript in WM266-4 cell lines expressing or not NR27 or RH28 nanobodies. After 18 hours and 24 hours post induction with doxycycline at 1µg/ml, no significant change in RHOA transcript level was observed. (B): RH28 expression in WM266.4 lentiviral cell line. Expression of RH28 was induced or not with doxycycline at 1µg/ml. After 20 hours of induction, cells were harvested and cleared cell lysates were incubated with Ni-NTA beads for 45 min. Endogenous RHOA and RAC1 proteins were revealed with corresponding antibodies and hs2dAb were revealed with myc-tag antibody. Dash line indicates a lane cropped. **(C)**: Similar immunoprecipitation experiment on cell line expressing RH28 or NR nanobodies upon doxycycline induction.

Supplementary Figure 1

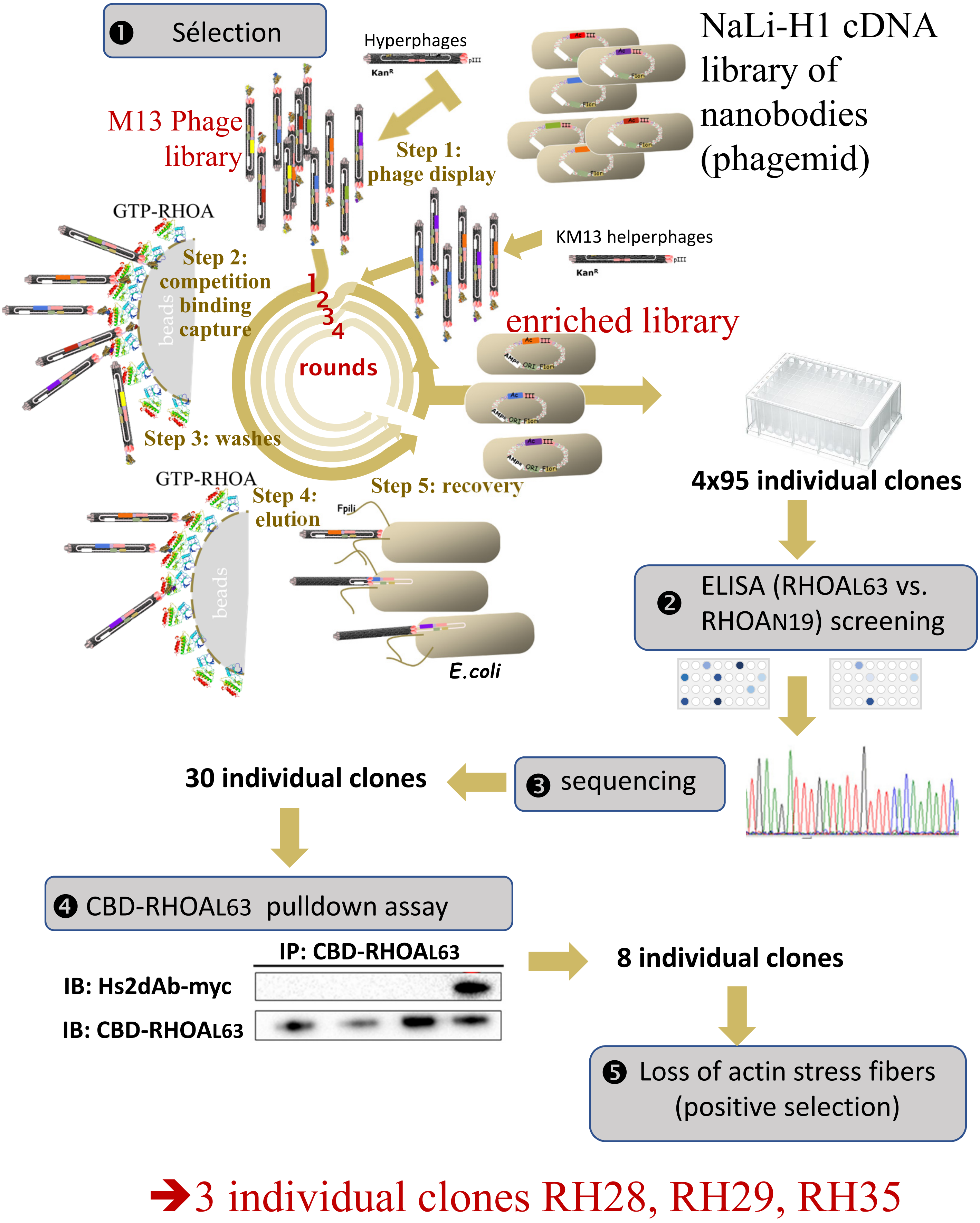

Supplementary Figure 2

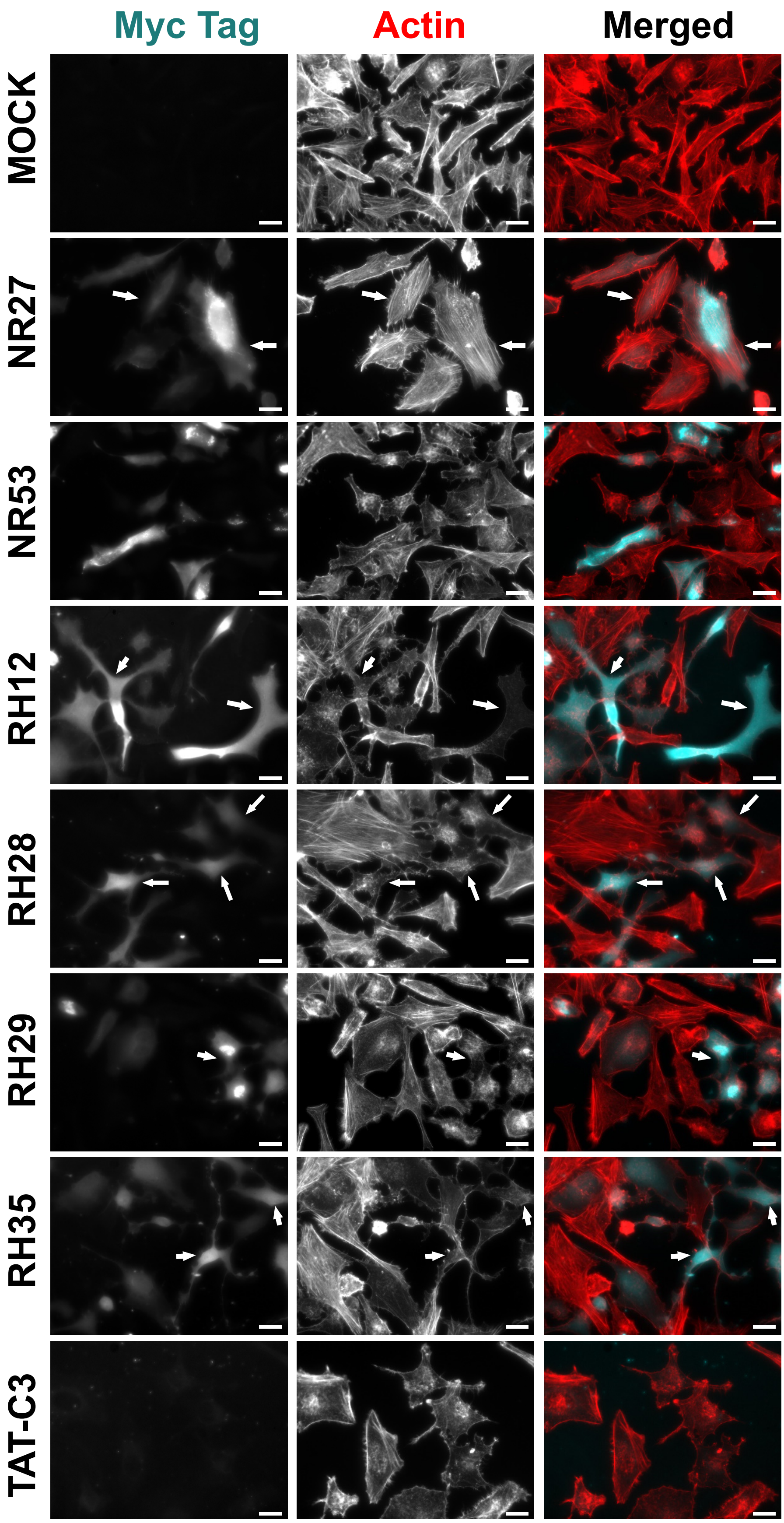

Supplementary Figure 3

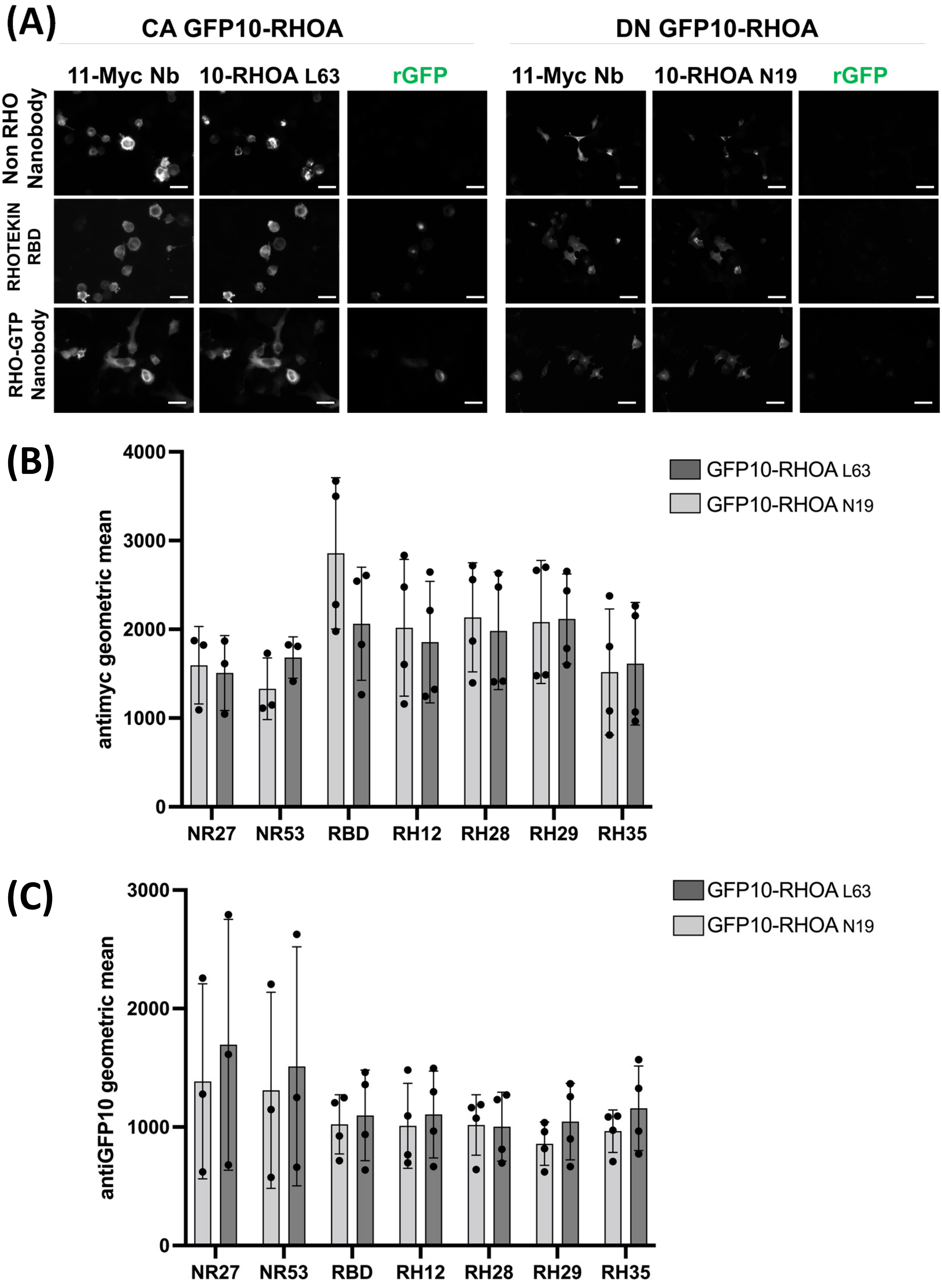

Supplementary Figure 4

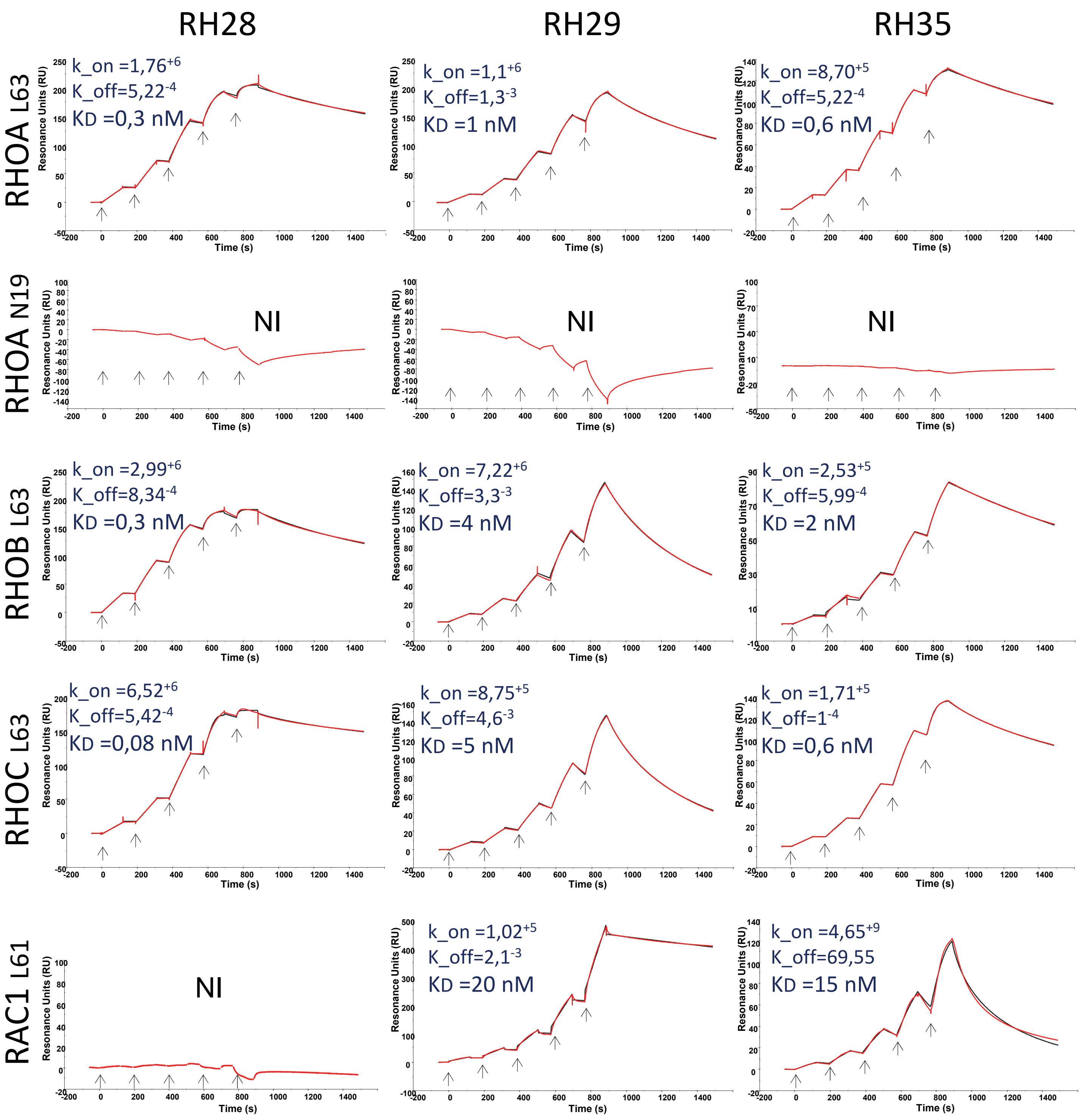

Supplementary Figure 5

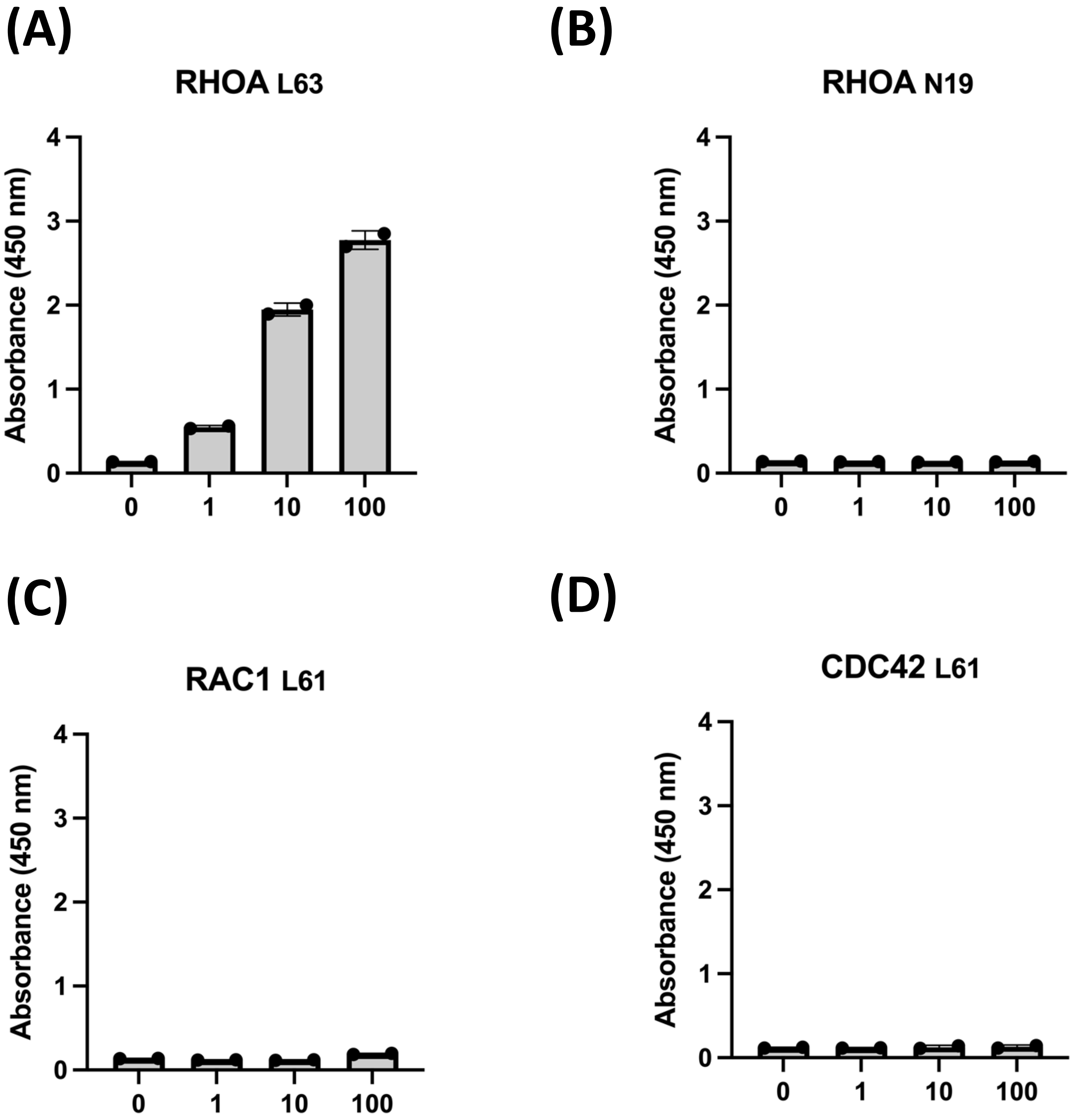

Supplementary Figure 6

(A)

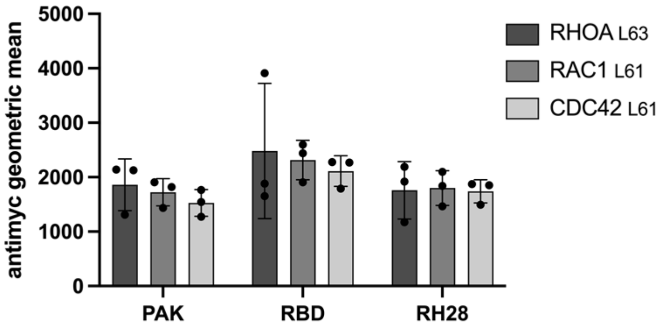

(B)

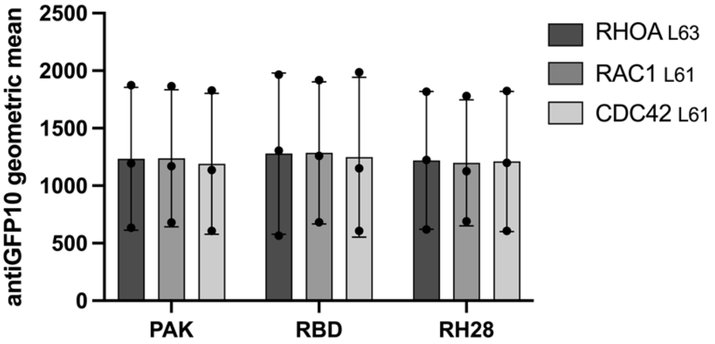

(C)

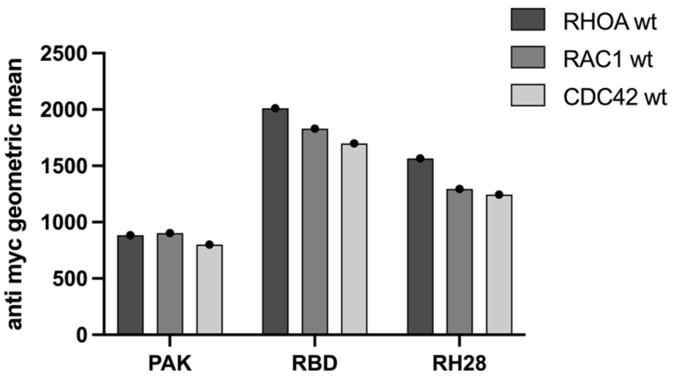

(D)

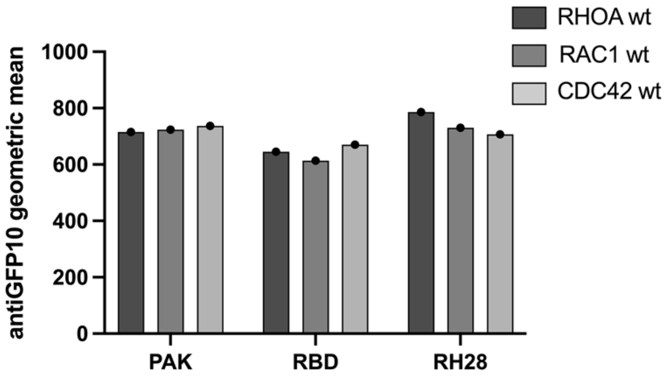

Supplementary Figure 7

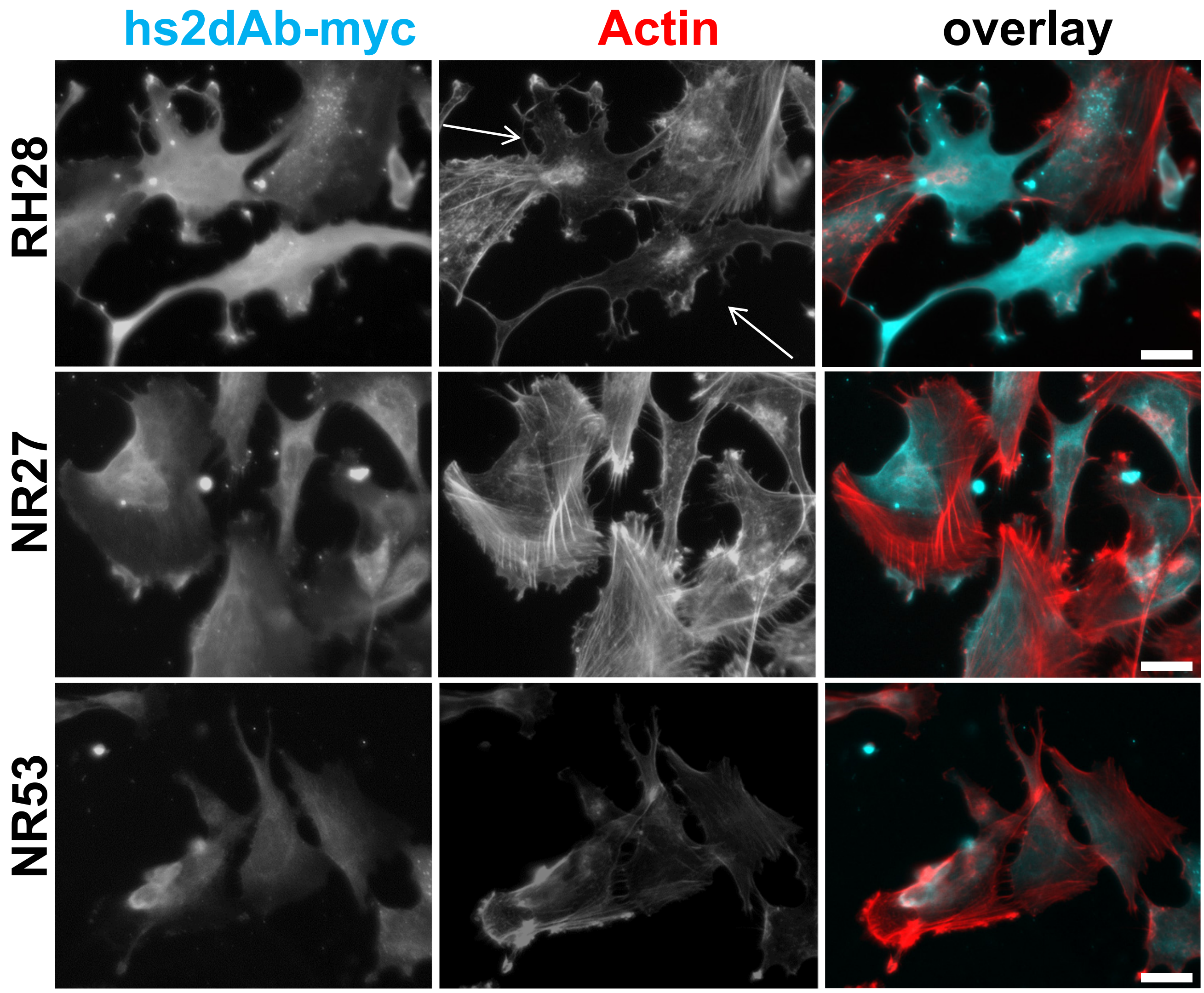

Supplementary Figure 8

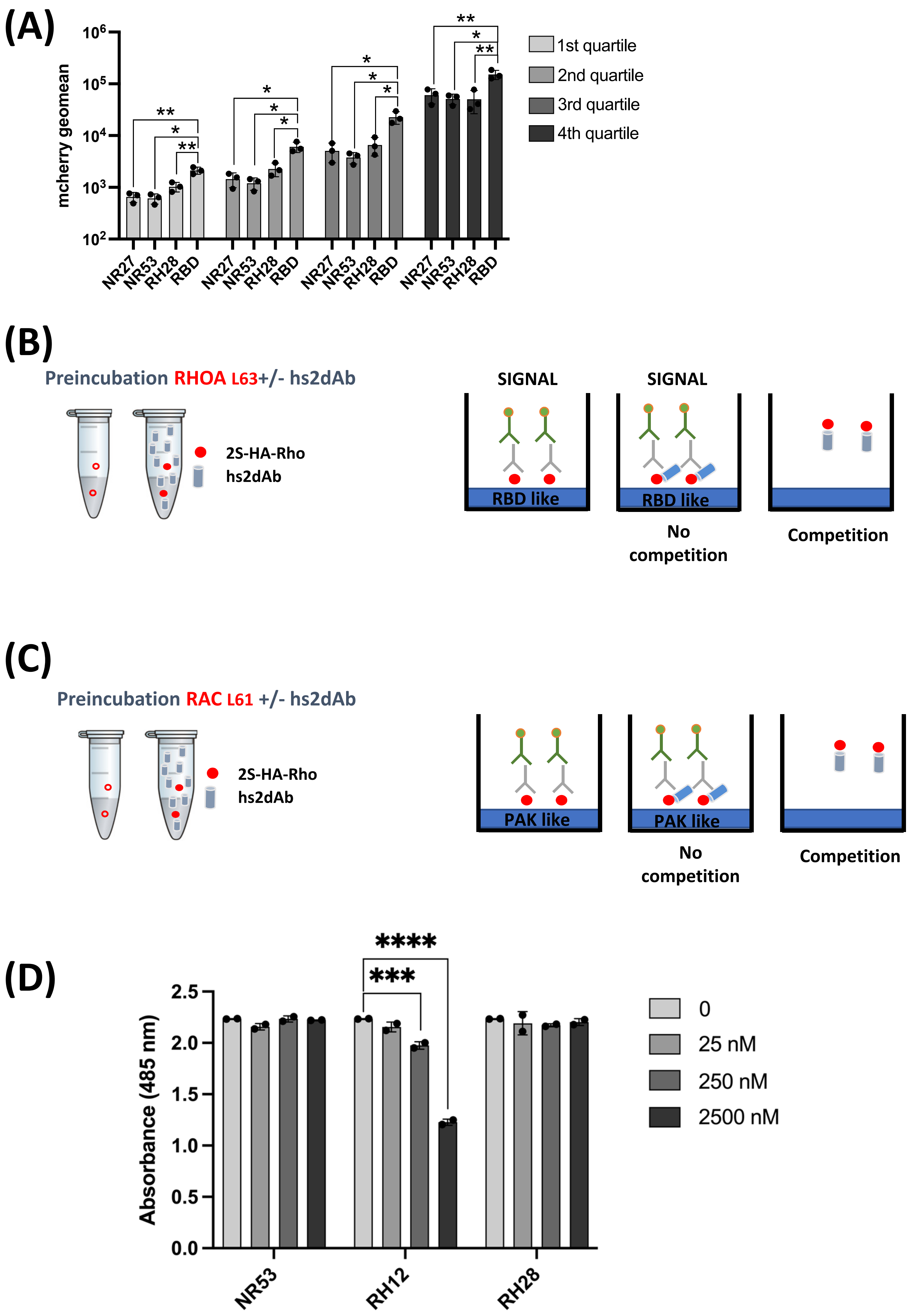

Supplementary Figure 9

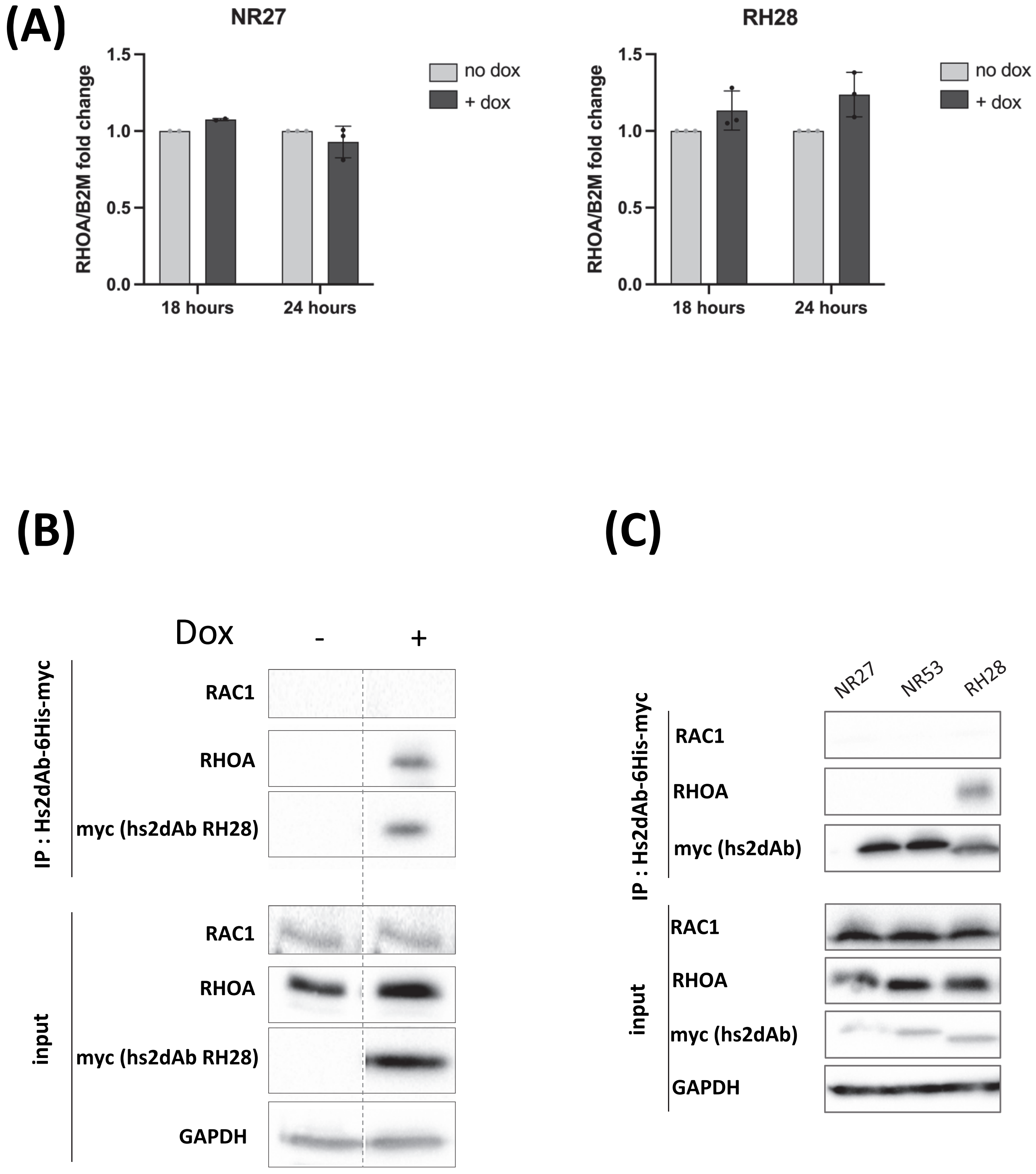

Supplement: Supplementary file 1 [file Presentation_1.pdf]
